# Supplementary material for: An in-situ peptide-antibody self-assembly to block CD47 and CD24 signaling enhances macrophage-mediated phagocytosis and anti-tumor immune responses
Source: Nat Commun. 2024 Jul 6;15:5670. doi: 10.1038/s41467-024-49825-6 (PMC11227529; doi:10.1038/s41467-024-49825-6)
Supplement: Supplementary file 1 — Supplementary Information [file 41467_2024_49825_MOESM1_ESM.pdf]

Supplementary Information

An in-situ peptide-antibody self-assembly to block CD47 and CD24 signaling enhances macrophage-mediated phagocytosis and anti-tumor immune responses

Weiqi Zhang[1, 2, 3], Yinghua Zeng[1, 3, 4], Qiuqun Xiao[3, 4], Yuanyuan Wu[5], Jiale Liu[3, 4], Haocheng Wang[6], Yuting Luo[2], Jie Zhan[7, 8, *], Ning Liao[2, *], Yanbin Cai[3, 4, 9, *]

[1]Guangdong Cardiovascular Institute, Guangdong Provincial People's Hospital, Guangdong Academy of Medical Science, Southern Medical University, Guangzhou, China

[2]Department of Breast Surgery, Guangdong Provincial People's Hospital, Guangdong Academy of Medical Sciences, Southern Medical University, Guangzhou, China

[3]Guangdong Provincial Biomedical Engineering Technology Research Center for Cardiovascular Disease, Zhujiang Hospital, Southern Medical University, Guangzhou, China

[4]Department of Cardiology and Laboratory of Heart Center, Zhujiang Hospital, Southern Medical University, Guangzhou, China

[5]Department of Hepatobiliary Surgery, Zhujiang Hospital, Southern Medical University, Guangzhou, China

[6]Department of Gastrointestinal Surgery, Zhujiang Hospital, Southern Medical University, Guangzhou, China

[7]Department of Laboratory Medicine, Nanfang Hospital, Southern Medical University, Guangzhou, China

[8]Guangdong Engineering and Technology Research Center for Rapid Diagnostic Biosensors, Nanfang Hospital, Southern Medical University, Guangzhou, China

[9]Department of Cardiovascular Surgery, Zhujiang Hospital, Southern Medical University, Guangzhou, China

Table of Contents

Supplementary Fig. 1 4
Supplementary Fig. 2 5
Supplementary Fig. 3 7
Supplementary Fig. 4 8
Supplementary Fig. 5 9
Supplementary Fig. 6 10
Supplementary Fig. 7 11
Supplementary Fig. 8 12
Supplementary Fig. 9 13
Supplementary Fig. 10 14
Supplementary Fig. 11 15
Supplementary Fig. 12 16
Supplementary Table 1 17
Supplementary Fig. 13 18
Supplementary Fig. 14 19
Supplementary Fig. 15 20
Supplementary Fig. 16 21
Supplementary Fig. 17 22
Supplementary Fig. 18 23
Supplementary Fig. 19 24
Supplementary Fig. 20 25
Supplementary Fig. 21 26
Supplementary Fig. 22 27
Supplementary Fig. 23 28
Supplementary Fig. 24 29
Supplementary Fig. 25 30
Supplementary Fig. 26 31
Supplementary Fig. 27 32

Supplementary Fig. 28 .... **33**

Supplementary Fig. 29 .... **35**

Supplementary Fig. 30 .... **36**

Supplementary Fig. 31 .... **37**

Supplementary Fig. 32 .... **38**

Supplementary Fig. 33 .... **39**

Supplementary Fig. 34 .... **40**

Supplementary Table 2 .... **41**

Supplementary Fig. 35 .... **42**

Supplementary Fig. 36 .... **43**

Supplementary Fig. 37 .... **44**

Supplementary Fig. 38 .... **45**

Supplementary Fig. 39 .... **46**

Supplementary Fig. 40 .... **47**

Supplementary Fig. 41 .... **49**

Supplementary Fig. 42 .... **50**

Supplementary Fig. 43 .... **51**

Supplementary Fig. 44 .... **52**

Supplementary Fig. 45 .... **53**

Supplementary Fig. 46 .... **54**

Supplementary Fig. 47 .... **55**

Supplementary Fig. 48 .... **56**

Supplementary Table 3 .... **57**

Supplementary References **58**

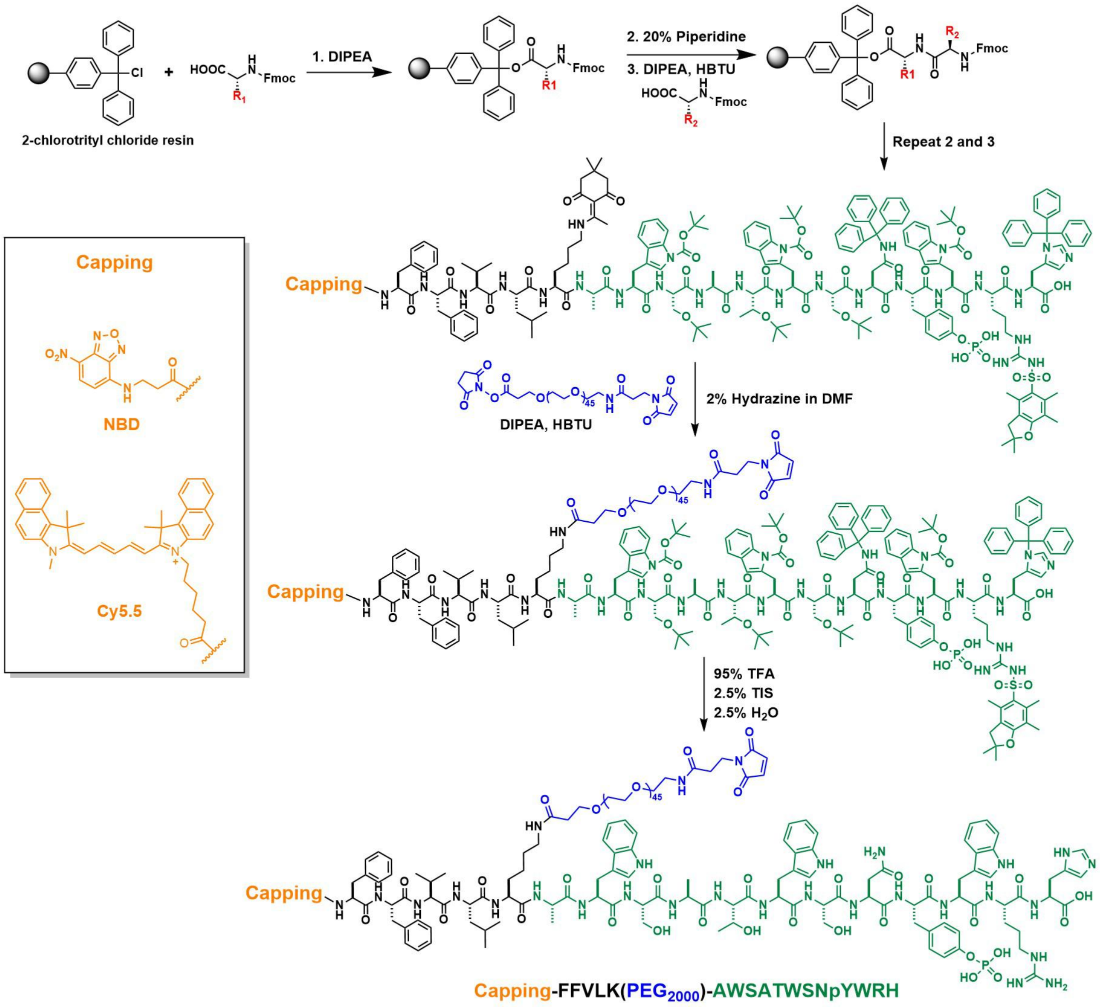

Supplementary Fig. 1 The synthetic route for Pep-PEG.

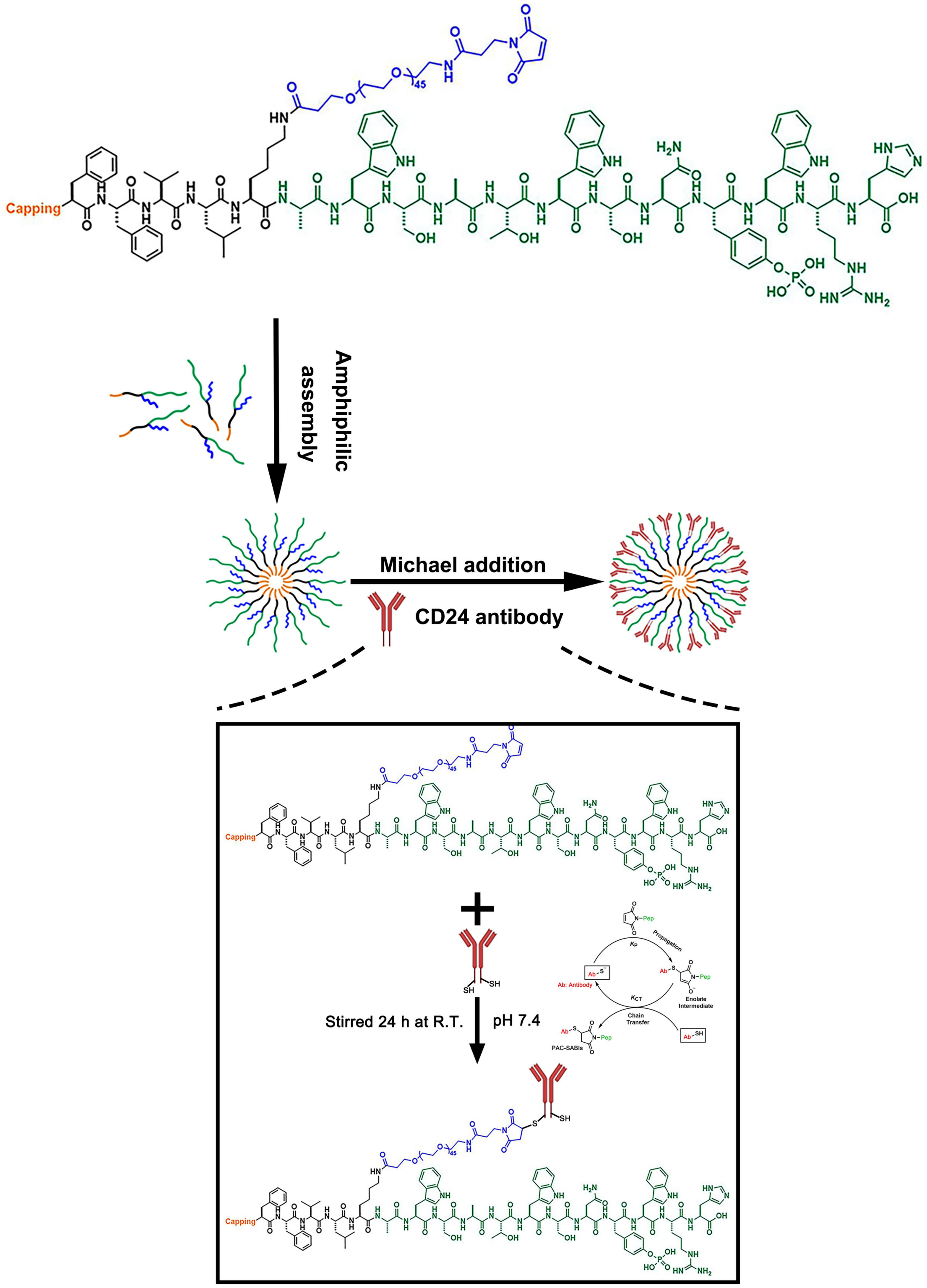

Supplementary Fig. 2 The synthetic route for PAC-SABIs by Michael addition reaction.

Reaction mechanism: thiol groups are typically situated in specific areas of antibody molecule, particularly within the Fc region. The sulfur atom in the thiol group, endowed with a lone pair of electrons, acted as a nucleophilic agent. Its nucleophilicity was directed towards maleimides,

characterized by their electron-deficient carbon-carbon double bonds, serving as electrophilic centers. The reaction was initiated with the sulfur atom of the thiol group attacking one of the carbon atoms of the maleimide's carbon-carbon double bond. Concurrently, the electrons from the double bond were shifted towards the other carbon atom. This attack resulted in the formation of an enolate intermediate, accompanied by the creation of a new Ab-S-C (Ab, Antibody) bond. The enolate intermediates thus formed were often not inherently stable and underwent a series of internal electronic rearrangements. Following the stabilization of the enolate intermediate, chain transfer occurred. At this point, the original double bond was completely opened, forming a new carbon-carbon single bond. The final stage involved the formation of the stable addition product post chain transfer and electron rearrangement. This product was characterized by the newly formed carbon-sulfur bond and a new carbon-carbon single bond, marking the completion of the Michael addition process and the formation of the stable compound.

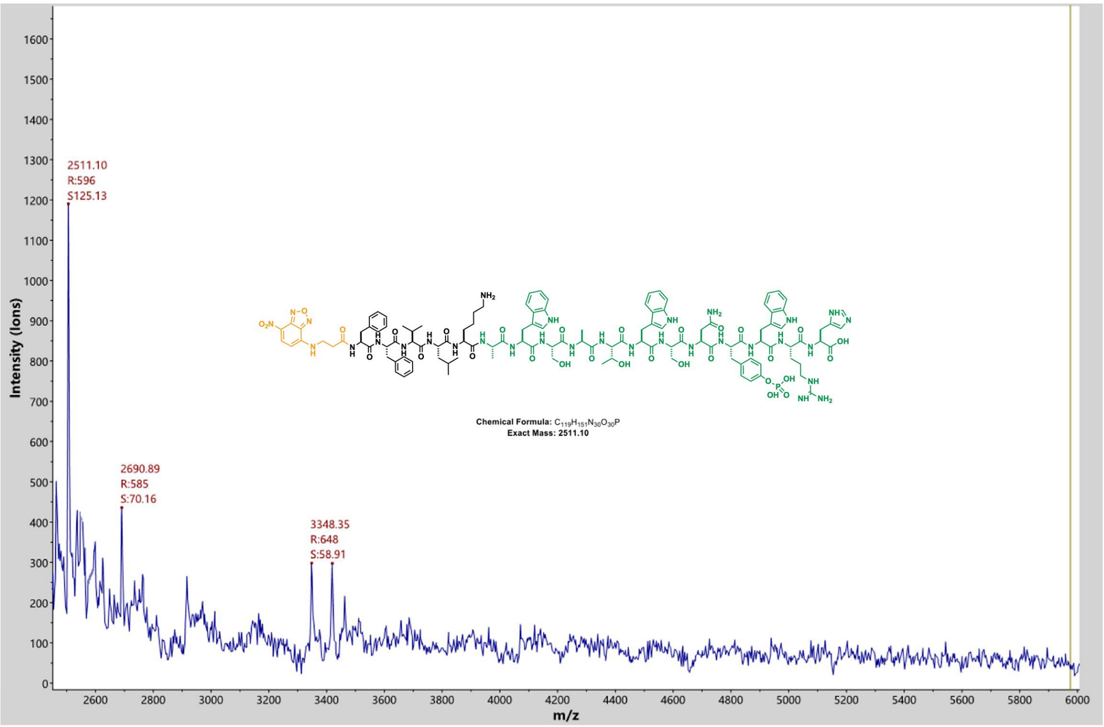

Supplementary Fig. 3 MALDI-TOF mass spectrometry of Pep (NBD).

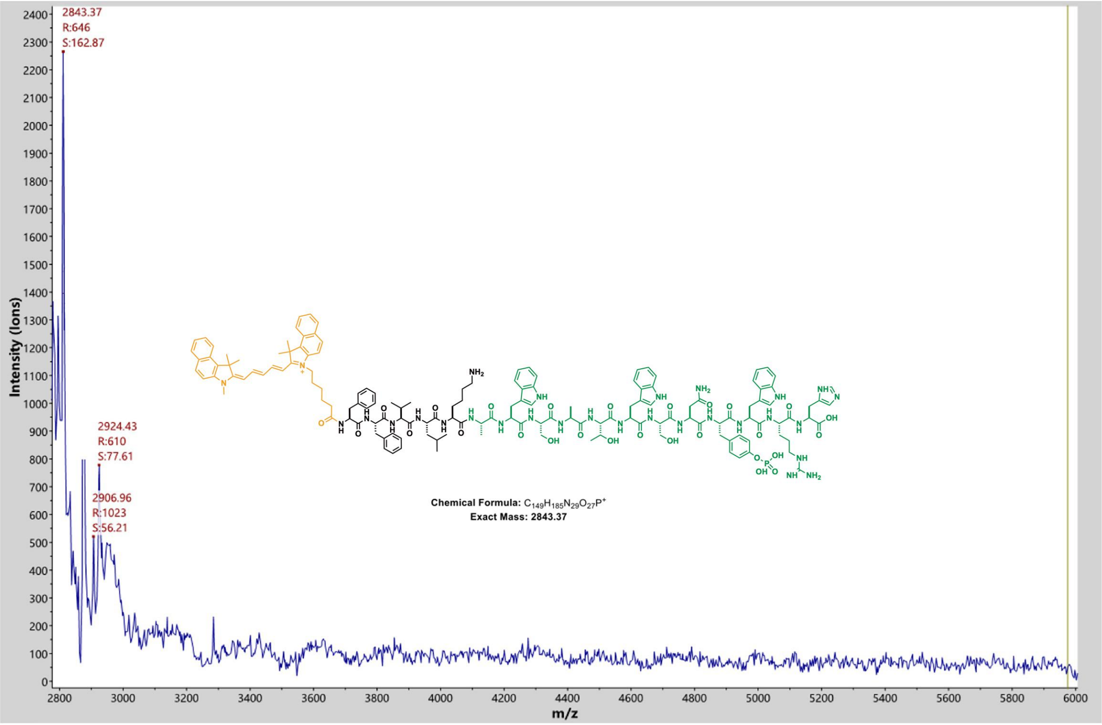

Supplementary Fig. 4 MALDI-TOF mass spectrometry of Pep (Cy5.5).

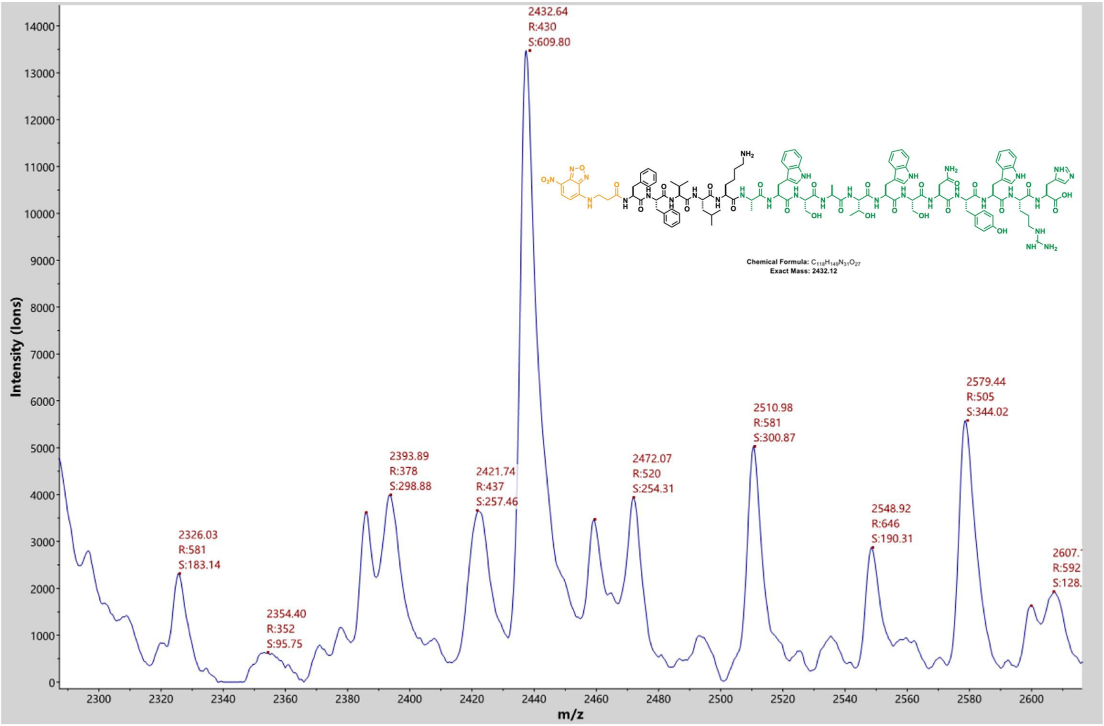

Supplementary Fig. 5 MALDI-TOF mass spectrometry of P2 (NBD).

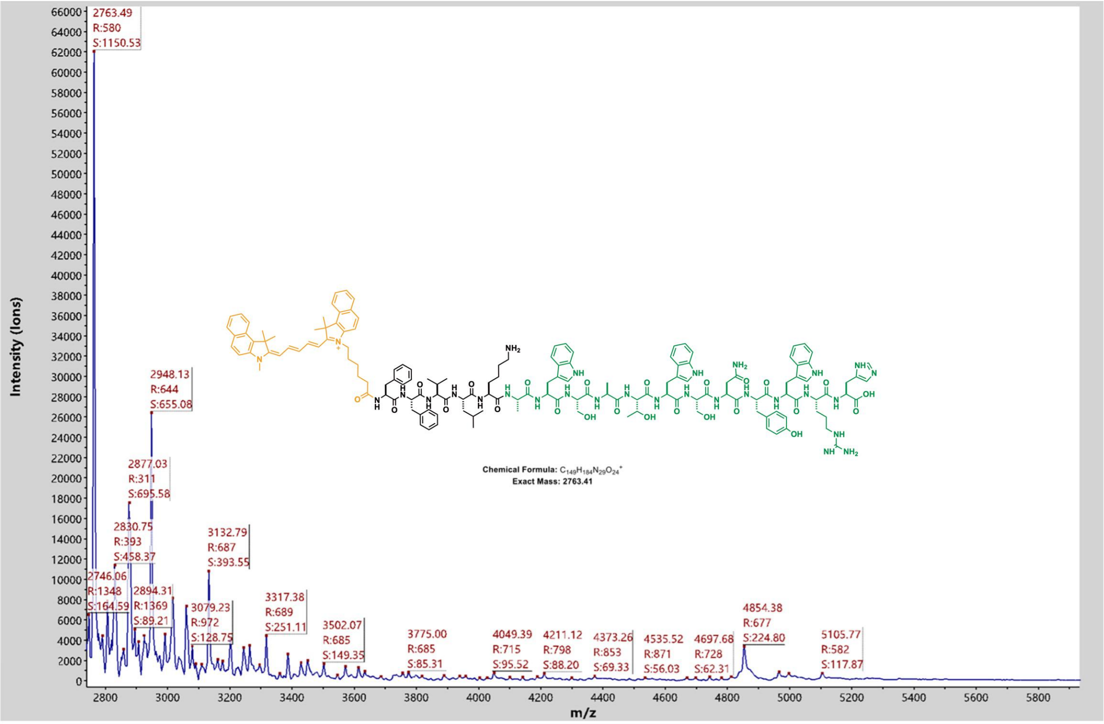

Supplementary Fig. 6 MALDI-TOF mass spectrometry of P2 (Cy5.5).

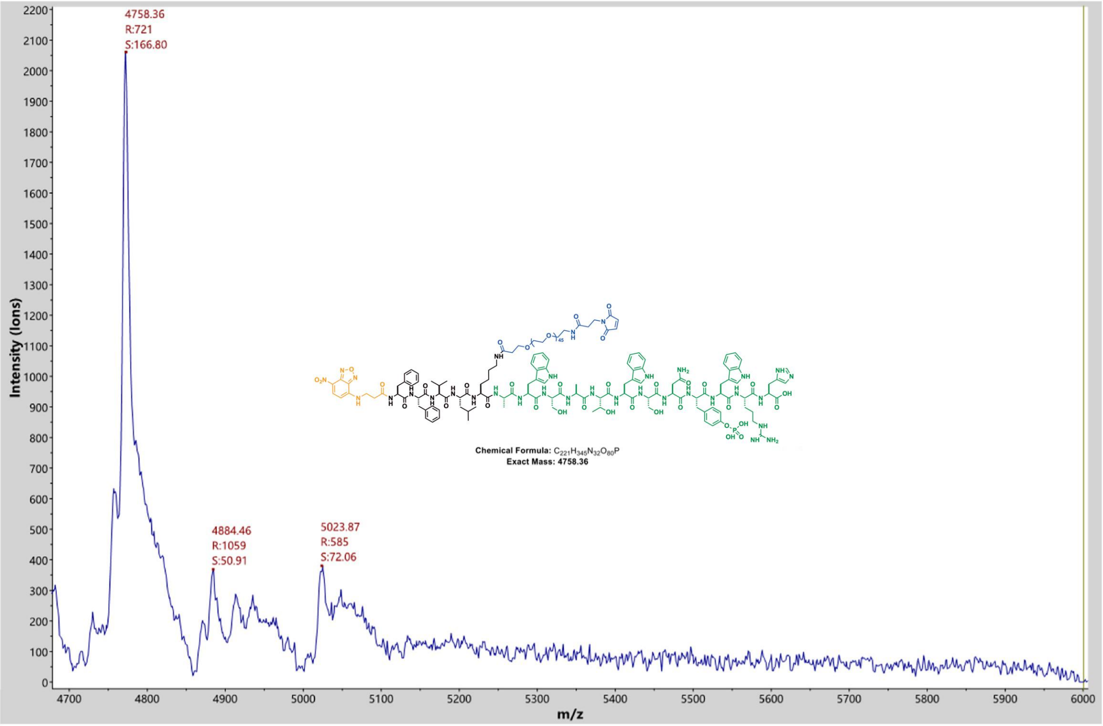

Supplementary Fig. 7 MALDI-TOF mass spectrometry of Pep-PEG (NBD).

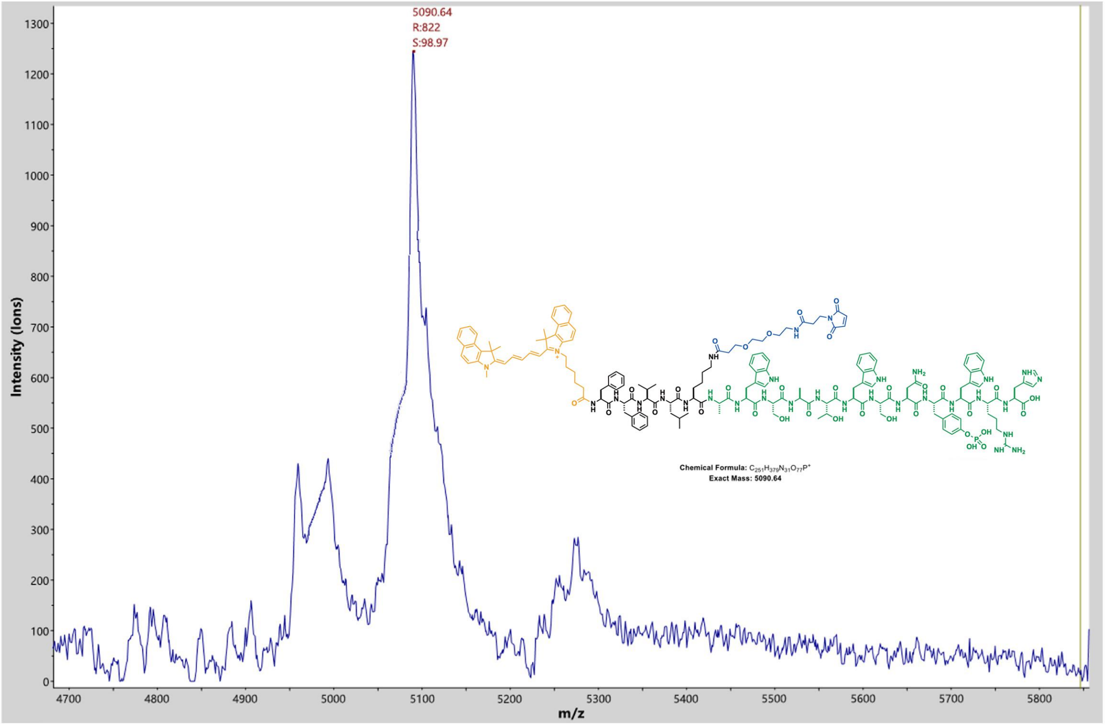

Supplementary Fig. 8 MALDI-TOF mass spectrometry of Pep-PEG (Cy5.5).

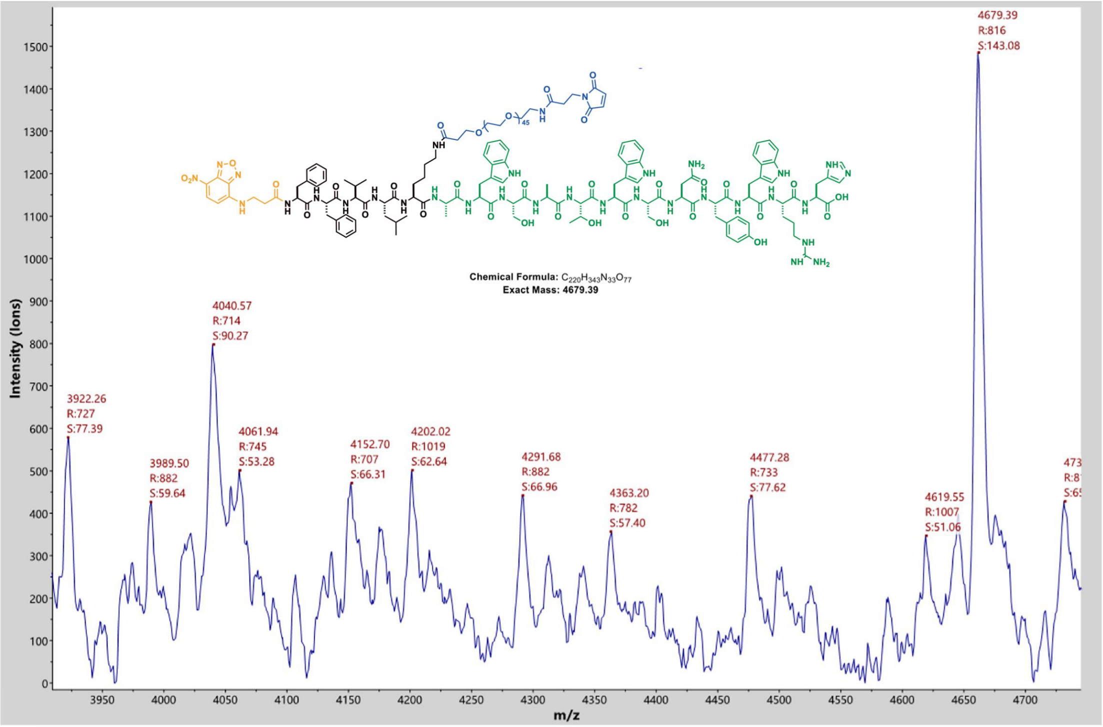

Supplementary Fig. 9 MALDI-TOF mass spectrometry of SAMIs (NBD).

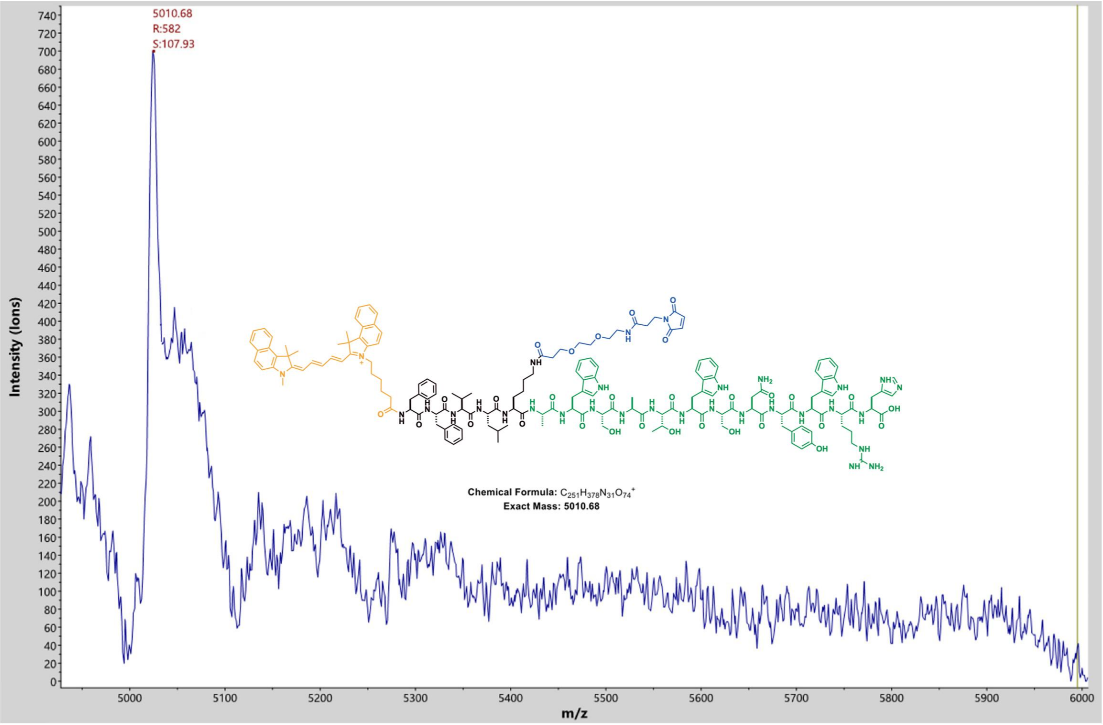

Supplementary Fig. 10 MALDI-TOF mass spectrometry of SAMI (Cy5.5).

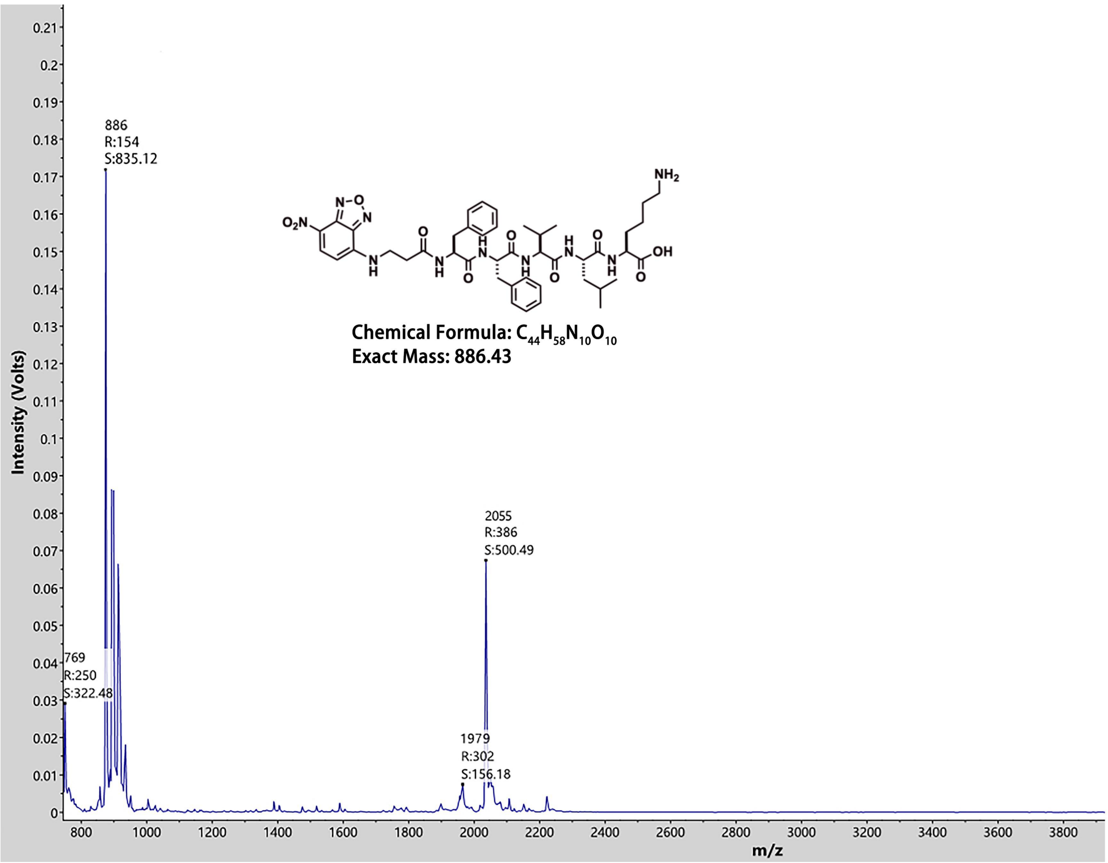

Supplementary Fig. 11 MALDI-TOF mass spectrometry of NBD-FFVLK.

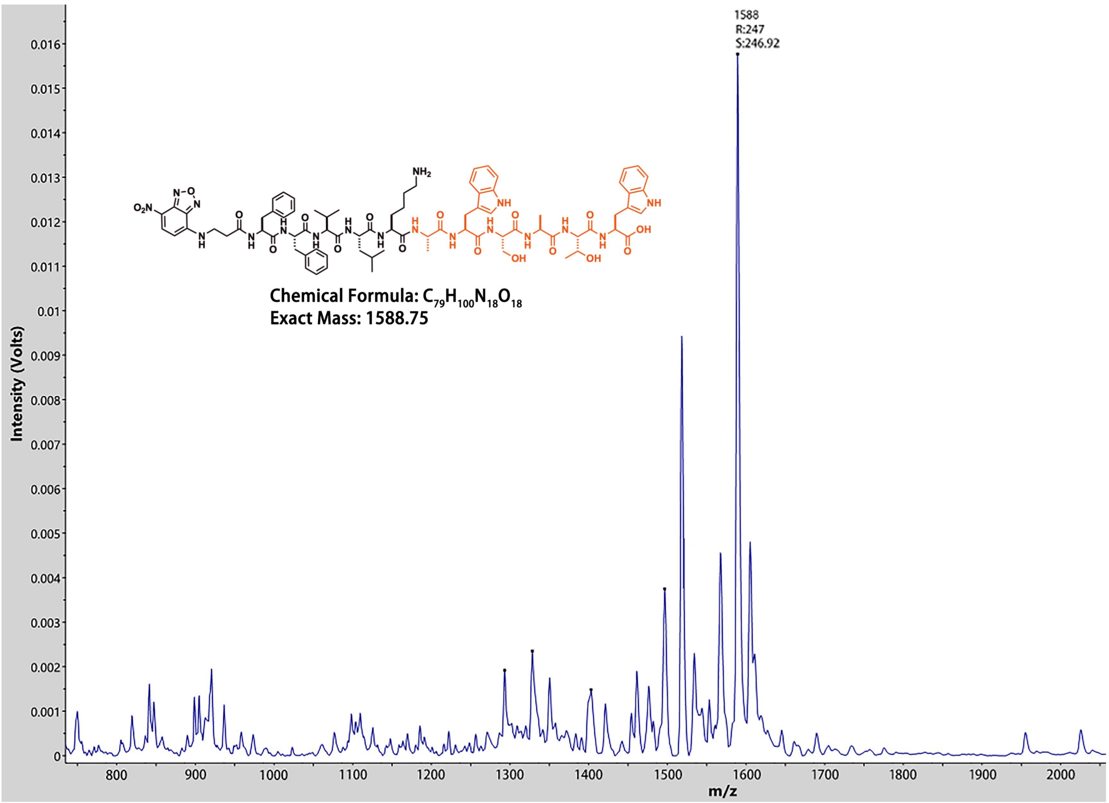

Supplementary Fig. 12 MALDI-TOF mass spectrometry of NBD-FFVLK-AWSATW.

Supplementary Table 1 Zeta potential of PAC-SABIs with different ratio of anti-CD24 mAb to Peptide.

mAb:peptide (w/w)	Zeta potential (mV)
1: 1	-14.3 ± 0.6
1: 5	-12.3 ± 0.7
1: 10	-11.7 ± 0.8
1: 20	-11.7 ± 1.2
1: 30	-11.5 ± 0.6
1: 40	-11.3 ± 0.4
1: 50	-11.1 ± 0.7

The Zeta potential was represented as mean ± SD (n = 3 independent experiments).

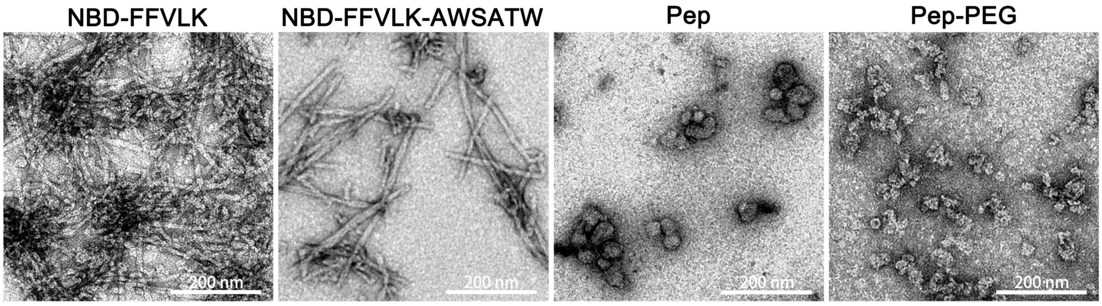

Supplementary Fig. 13 TEM images of NBD-FFVLK, NBD-FFVLK-AWSATW, Pep, Pep-PEG. Scale bar: 200 nm. Three independent experiments were performed.

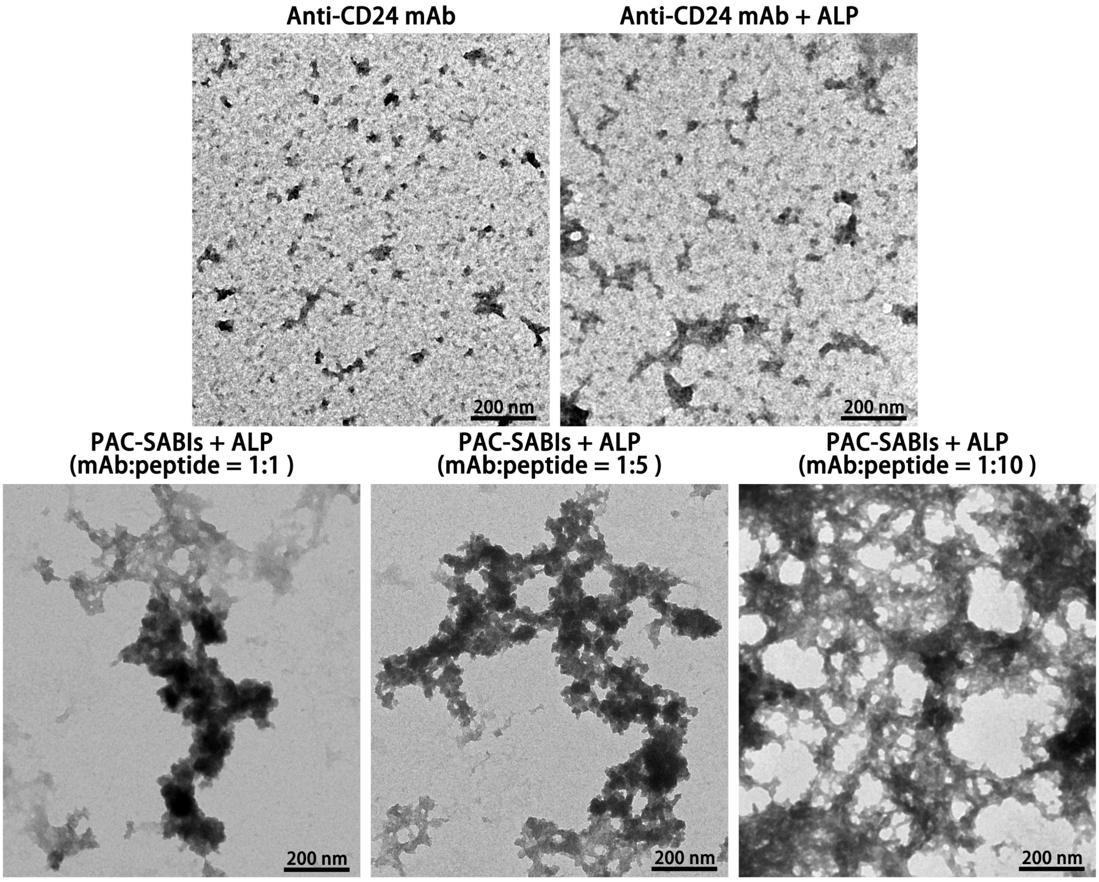

Supplementary Fig. 14 TEM images of anti-CD24 mAb, anti-CD24 mAb plus ALP, PAC-SABIs plus ALP (mAb: peptide = 1: 1), PAC-SABIs plus ALP (mAb: peptide = 1: 5), PAC-SABIs plus ALP (mAb: peptide = 1: 10). Scale bar: 200 nm. Three independent experiments were performed.

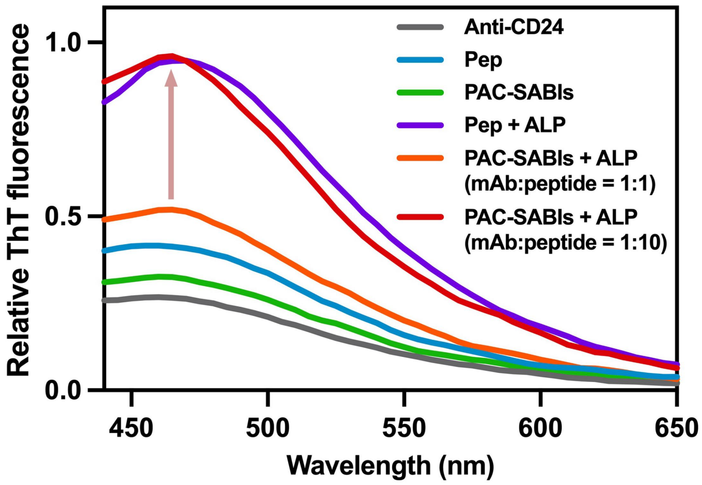

Supplementary Fig. 15 ThT fluorescence spectrum of anti-CD24 mAb, Pep, PAC-SABIs, Pep plus ALP, PAC-SABIs plus ALP (mAb: peptide = 1: 1), PAC-SABIs plus ALP (mAb: peptide = 1: 10). Three independent experiments were performed. Source data are provided as a Source Data file.

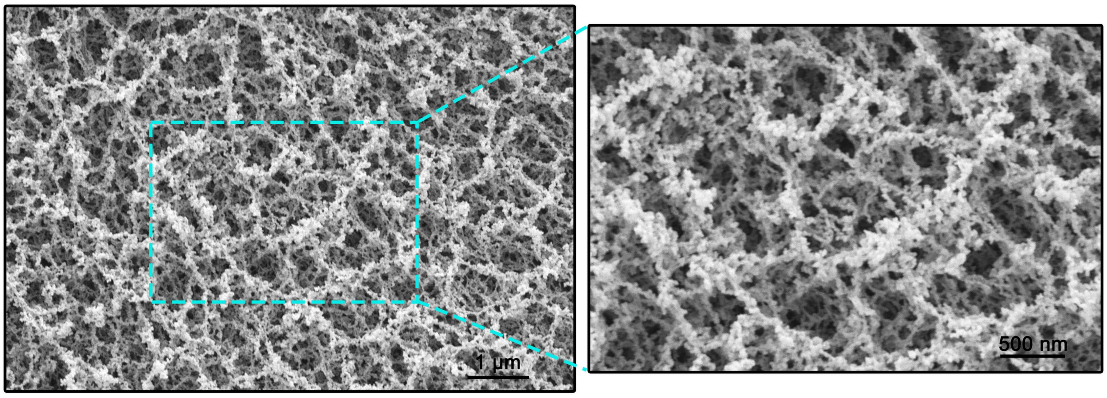

Supplementary Fig. 16 Representative SEM images of PAC-SABIs' microscopic structure. Scale bars are marked in the figures. Three independent experiments were performed.

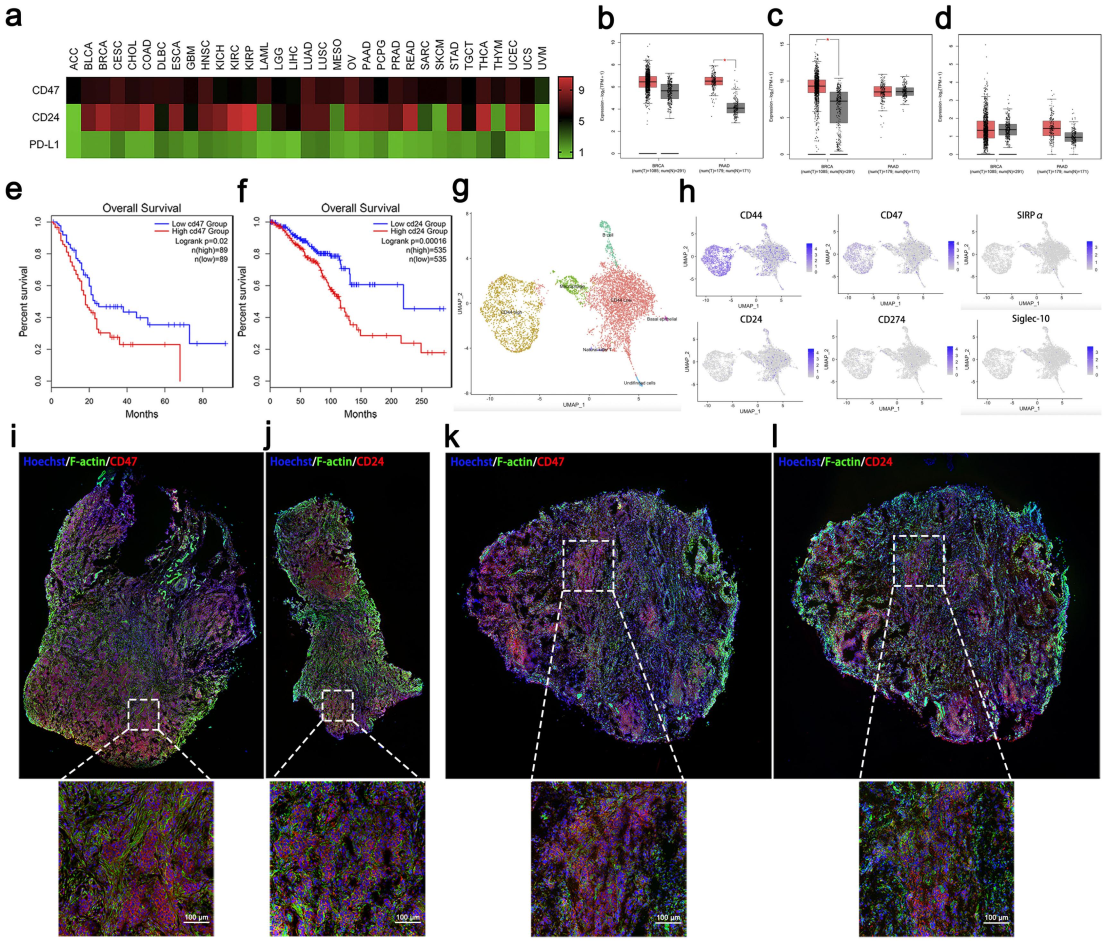

Supplementary Fig. 17 Expression of CD47 and CD24 in BC and PC. **a** Heatmap showing the normalized expression of CD47, CD24, and PD-L1 in a pan-cancer cohort. **b-d** Expression of CD47, CD24 and PD-L1 in BC and PC compared to matched normal tissue by analyzing GEPIA2[1] (*$p < 0.05$; the p value was analyzed by a two-tailed unpaired Student's t-test). **e** Overall survival for PC patients (n = 178) with high versus low CD47 expression as defined by median (Kaplan-Meier survival analysis was utilized and the p value was analyzed by log-rank test). **f** Overall survival for BC patients (n = 1070) with high versus low CD24 expression as defined by median (Kaplan-Meier survival analysis was utilized and the p value was analyzed by log-rank test). **g** UMAP dimension 1 and 2 plots displaying cells from a primary sample of BC, and cells colored by cluster identity (n = 20000 single cells). **h** CD44, CD47, CD24, CD274, SIRPα, and Siglec-10 expression overlaid onto UMAP space. **i, j** IF analysis of CD47 and CD24 in Patient 1. **k, l** IF analysis of CD47 and CD24 in Patient 2.

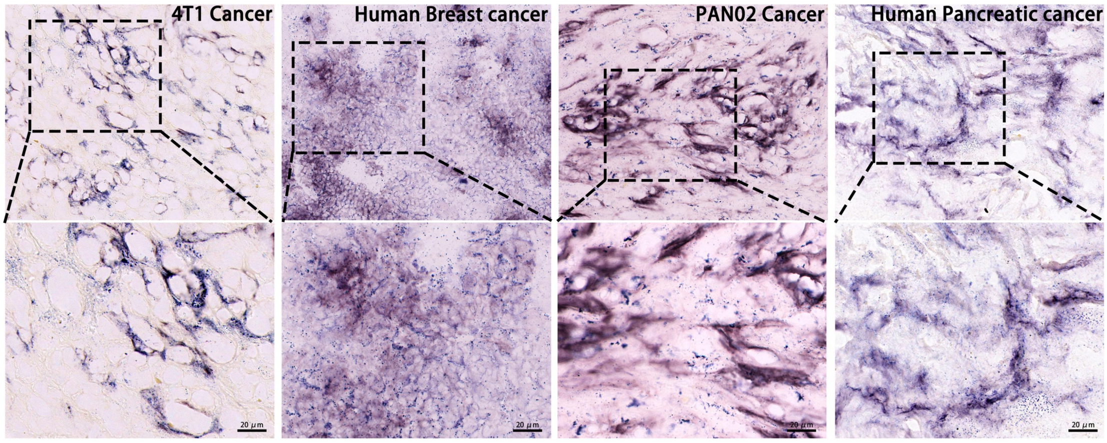

Supplementary Fig. 18 The expression of ALP in tissue sections of 4T1, human BC, PAN02, and human PC was detected by using the BCIP/NBT color development kit, as per manufacturer's instructions. Three independent experiments were performed. Scale bar: 20 μm.

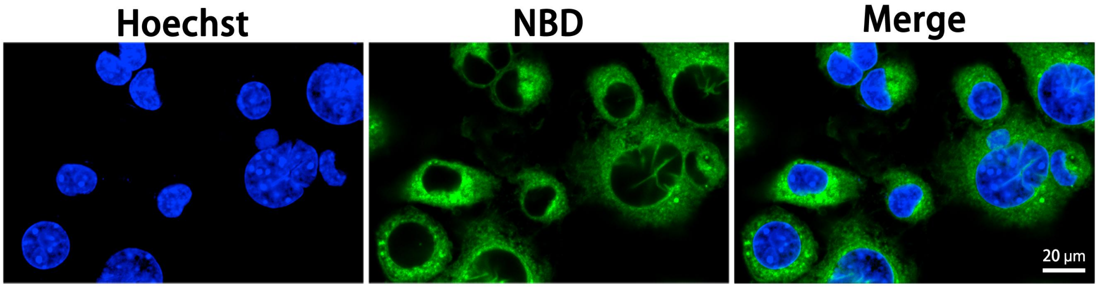

Supplementary Fig. 19 CLSM images of 4T1 cells treated with NBD-labeled SAMIs for 120 min. Scale bar: 20 μm. Three independent experiments were performed.

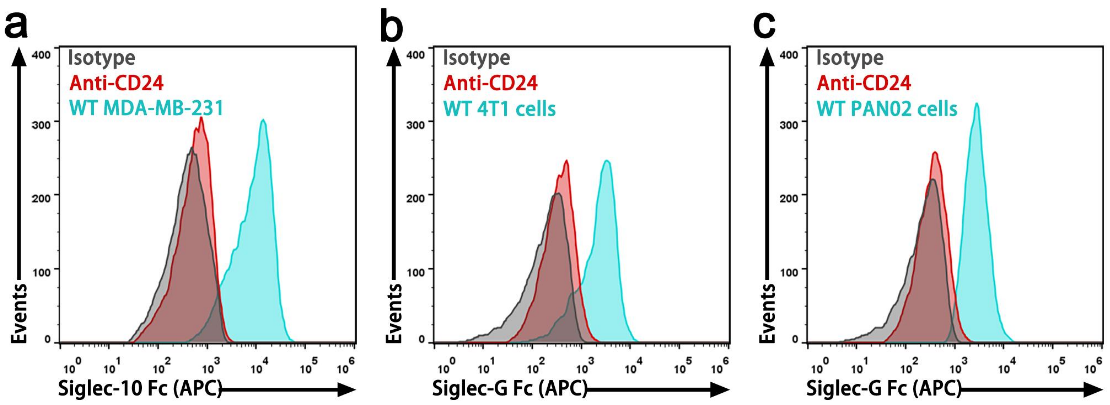

Supplementary Fig. 20 Flow cytometry histogram measuring binding of Siglec-10 to cancer cells. a Flow cytometry histogram measuring binding of Siglec-10 to MDA-MB-231 cells after different treatments. Three independent experiments were performed. **b** Flow cytometry histogram measuring binding of Siglec-G to 4T1 cells after different treatments. Three independent experiments were performed. **c** Flow cytometry histogram measuring binding of Siglec-G to PAN02 cells after different treatments. Three independent experiments were performed.

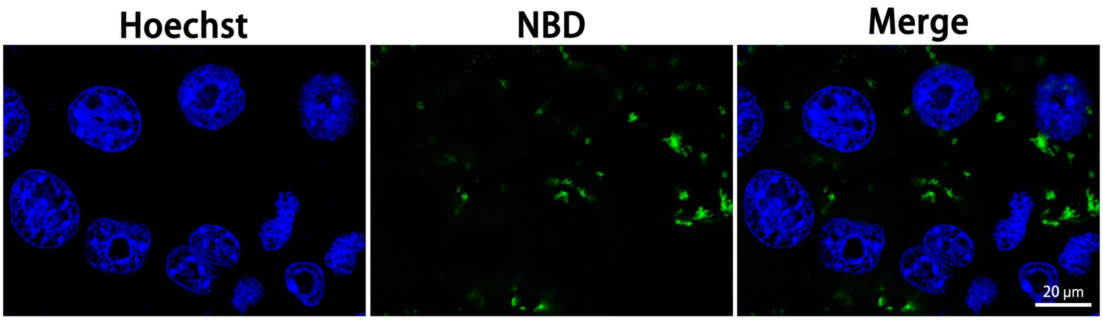

Supplementary Fig. 21 CLSM images of anti-CD24 mAb pre-blocked 4T1 cells treated with NBD-labeled PAC-SABIs for 120 min. Scale bar: 20 μm. Three independent experiments were performed.

a b c d

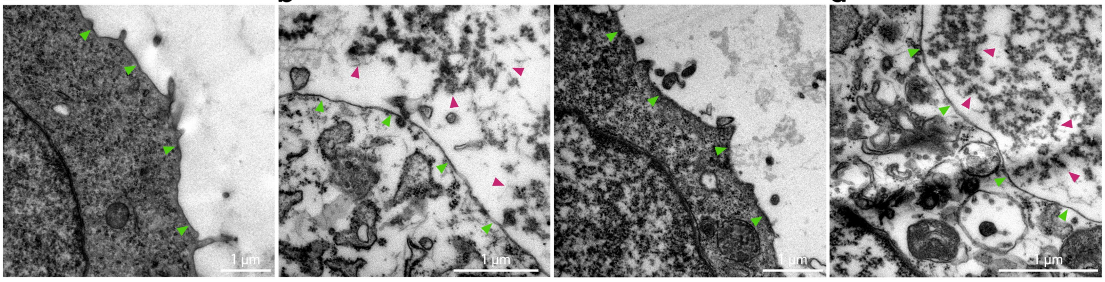

Supplementary Fig. 22 TEM images of cancer cells. a TEM images of untreated 4T1 cell. **b** TEM images of PAC-SABIs-treated 4T1 cell. **c** TEM images of untreated PAN02 cell. **d** TEM images of PAC-SABIs-treated PAN02 cell. The green arrows point to cell membrane; the red arrows point to PAC-SABIs. Scale bar: 1 μm. Three independent experiments were performed for Supplementary Fig. 22a-d.

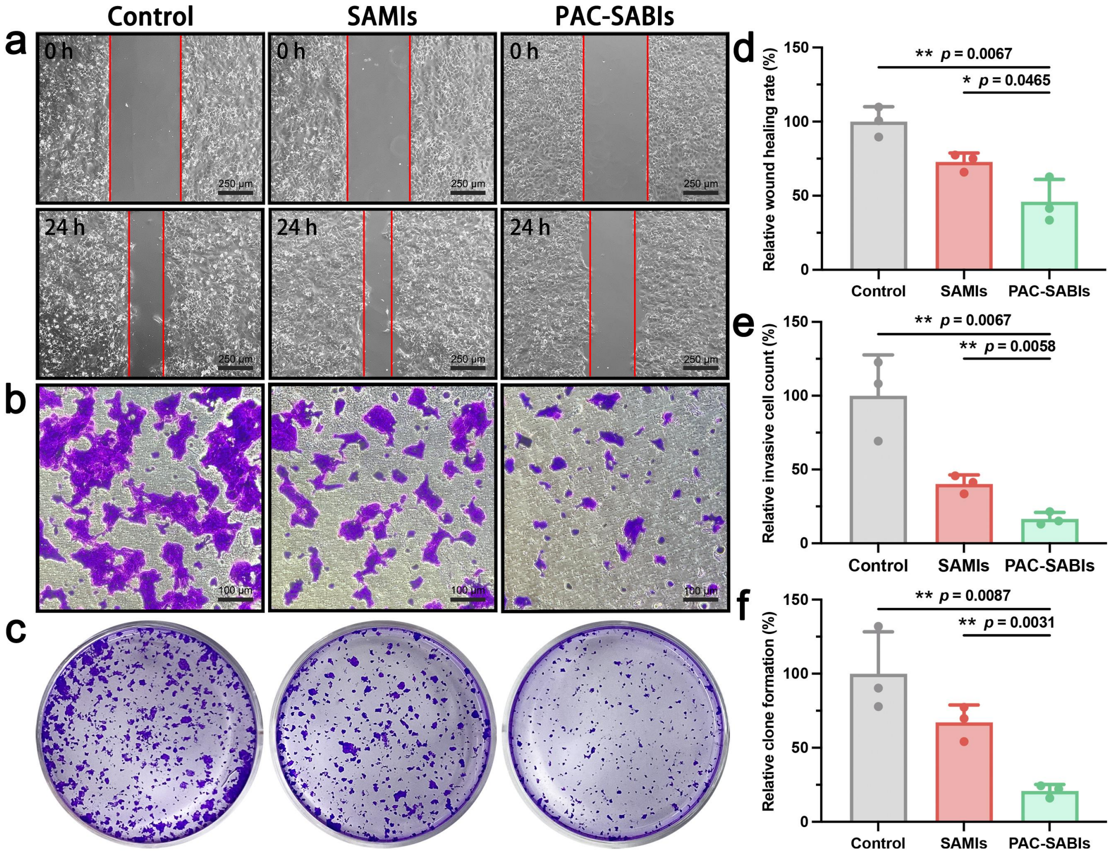

Supplementary Fig. 23 Wound healing, cell invasion, and Clone formation assays of 4T1 cells. a Representative microscopy image of a wound healing experiment of 4T1 cells treated with PBS, SAMIs and PAC-SABIs. Red areas indicate no migrated cells. Scale bar: 250 μm. **b** Representative microscopy images of migrated 4T1 cells treated with PBS, SAMIs and PAC-SABIs in a Transwell invasion assay. Scale bar: 100 μm. **c** Representative bright field images of 4T1 cell Clone formation. **d** Quantitative analysis of the wound healing rate. The error bars represent the mean ± SD (n = 3 independent experiments; $^{*}p < 0.05$, $^{**}p < 0.01$; the p value was analyzed by a two-tailed unpaired Student's t-test). **e** Quantitative analysis of the invasion rate. The error bars represent the mean ± SD (n = 3 independent experiments; $^{**}p < 0.01$; the p value was analyzed by a two-tailed unpaired Student's t-test). **f** Quantitative analysis of Clone formation rate. The error bars represent the mean ± SD (n = 3 independent experiments; $^{**}p < 0.01$; the p value was analyzed by a two-tailed unpaired Student's t-test). Source data are provided as a Source Data file.

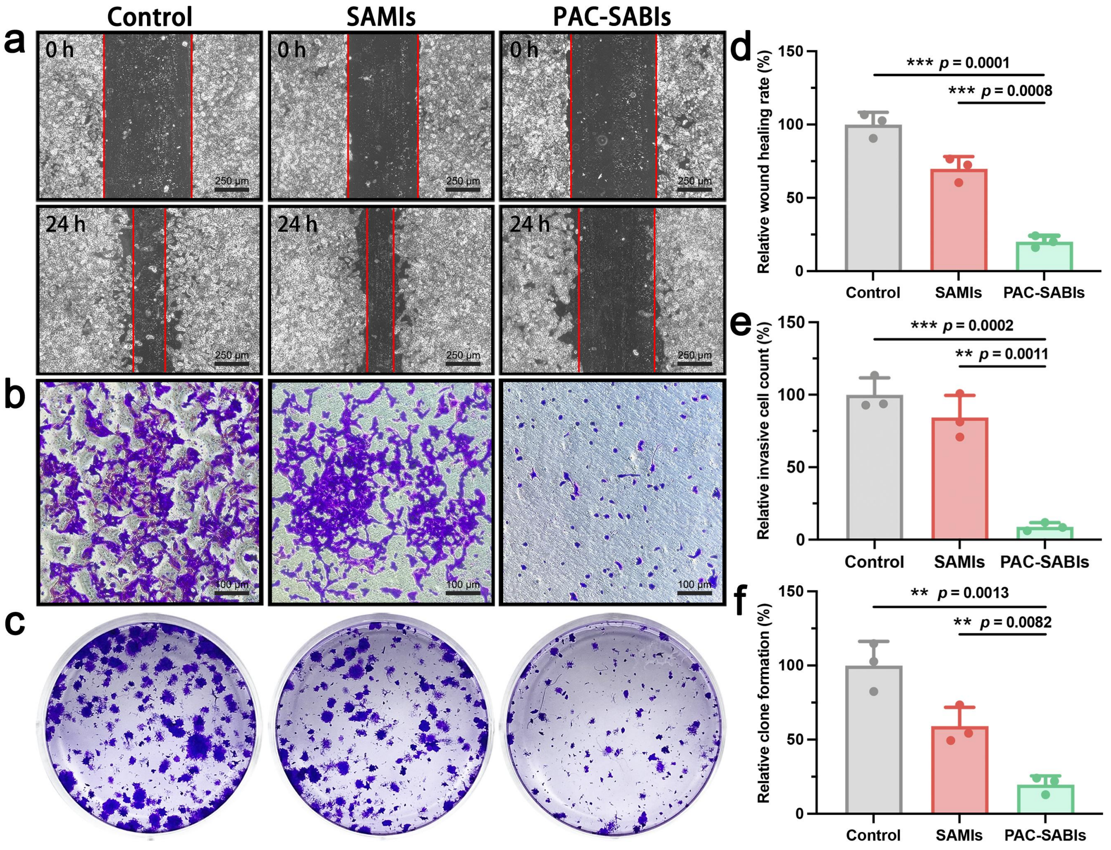

Supplementary Fig. 24 Wound healing, cell invasion, and Clone formation assays of PAN02 cells. a Representative microscopy image of a wound healing experiment of PAN02 cells treated with PBS, SAMIs and PAC-SABIs. Red areas indicate no migrated cells. Scale bar: 250 μm. **b** Representative microscopy images of migrated PAN02 cells treated with PBS, SAMIs and PAC-SABIs in a Transwell invasion assay. Scale bar: 100 μm. **c** Representative bright field images of PAN02 cell Clone formation. **d** Quantitative analysis of the wound healing rate. The error bars represent the mean ± SD (n = 3 independent experiments; $^{***}p < 0.001$; the p value was analyzed by a two-tailed unpaired Student's t-test). **e** Quantitative analysis of the invasion rate. The error bars represent the mean ± SD (n = 3 independent experiments; $^{**}p < 0.01$, $^{***}p < 0.001$; the p value was analyzed by a two-tailed unpaired Student's t-test). **f** Quantitative analysis of Clone formation rate. The error bars represent the mean ± SD (n = 3 independent experiments; $^{**}p < 0.01$; the p value was analyzed by a two-tailed unpaired Student's t-test). Source data are provided as a Source Data file.

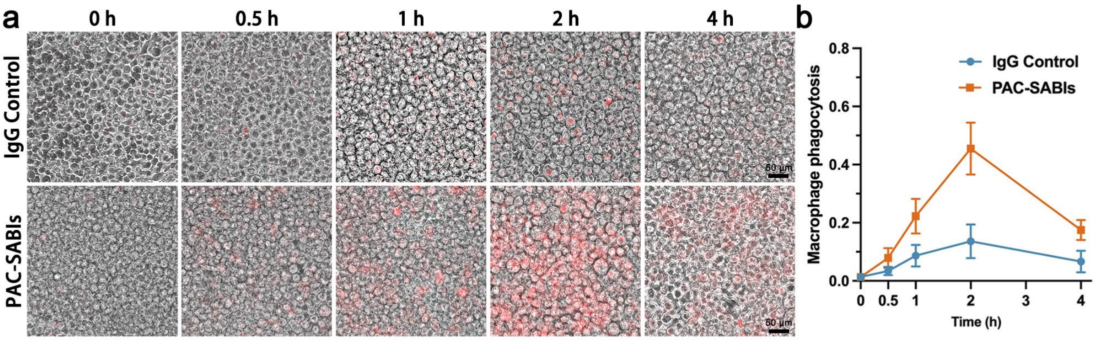

Supplementary Fig. 25 Promotion of phagocytic clearance of cancer cells via PAC-SABIs treatment in vitro. a Phagocytosis images of pHrodo-red^{+} over time. Scale bar: 50 μm. **b** Phagocytosis of PAN02 cells, in the presence of IgG control or PAC-SABIs. The error bars represent the mean ± SD (n = 3 independent experiments). Source data are provided as a Source Data file.

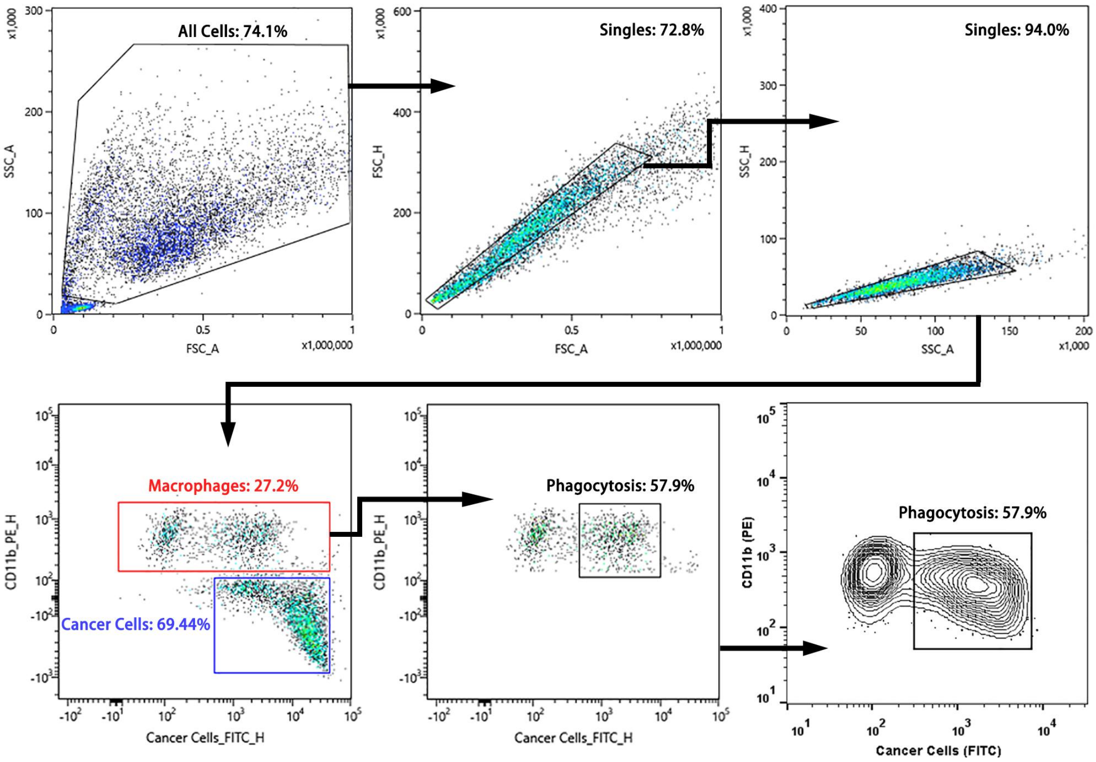

Supplementary Fig. 26 Gating strategy used for the evaluation of in vitro macrophage phagocytosis.

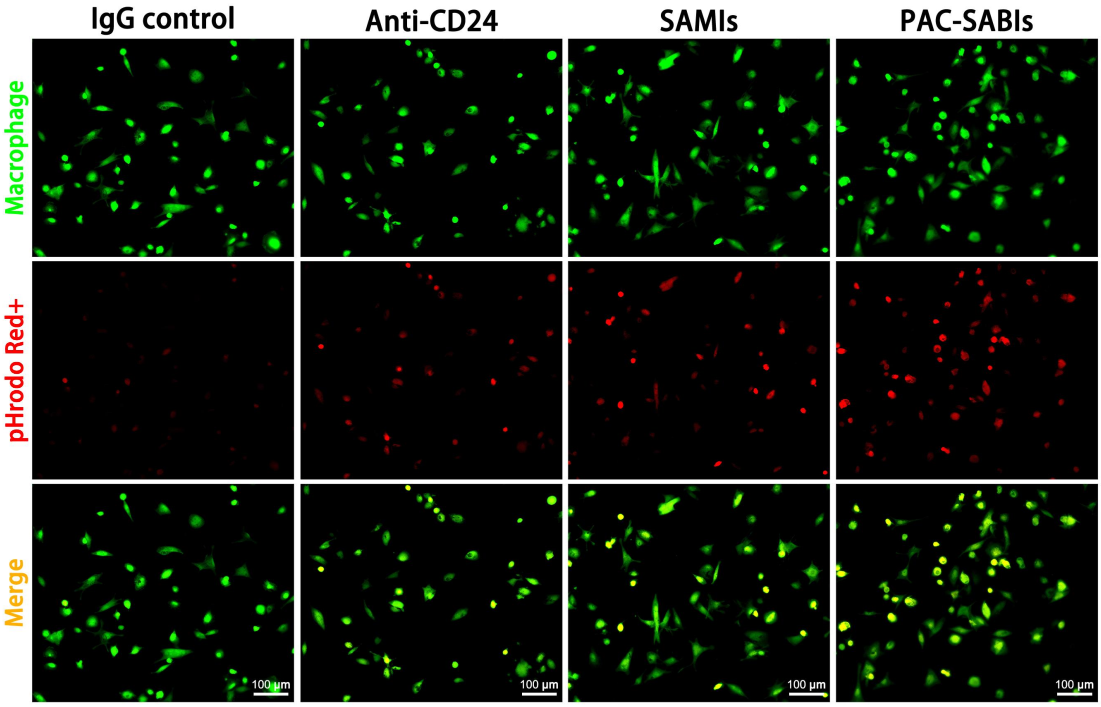

Supplementary Fig. 27 Representative CLSM images of in vitro phagocytosis of MDA-MB-231 cells (pHrodo-red^{+}, red) by human THP-1 monocytes derived macrophages (Calcein-AM; green). Scale bar: 100 μm. Three independent experiments were performed.

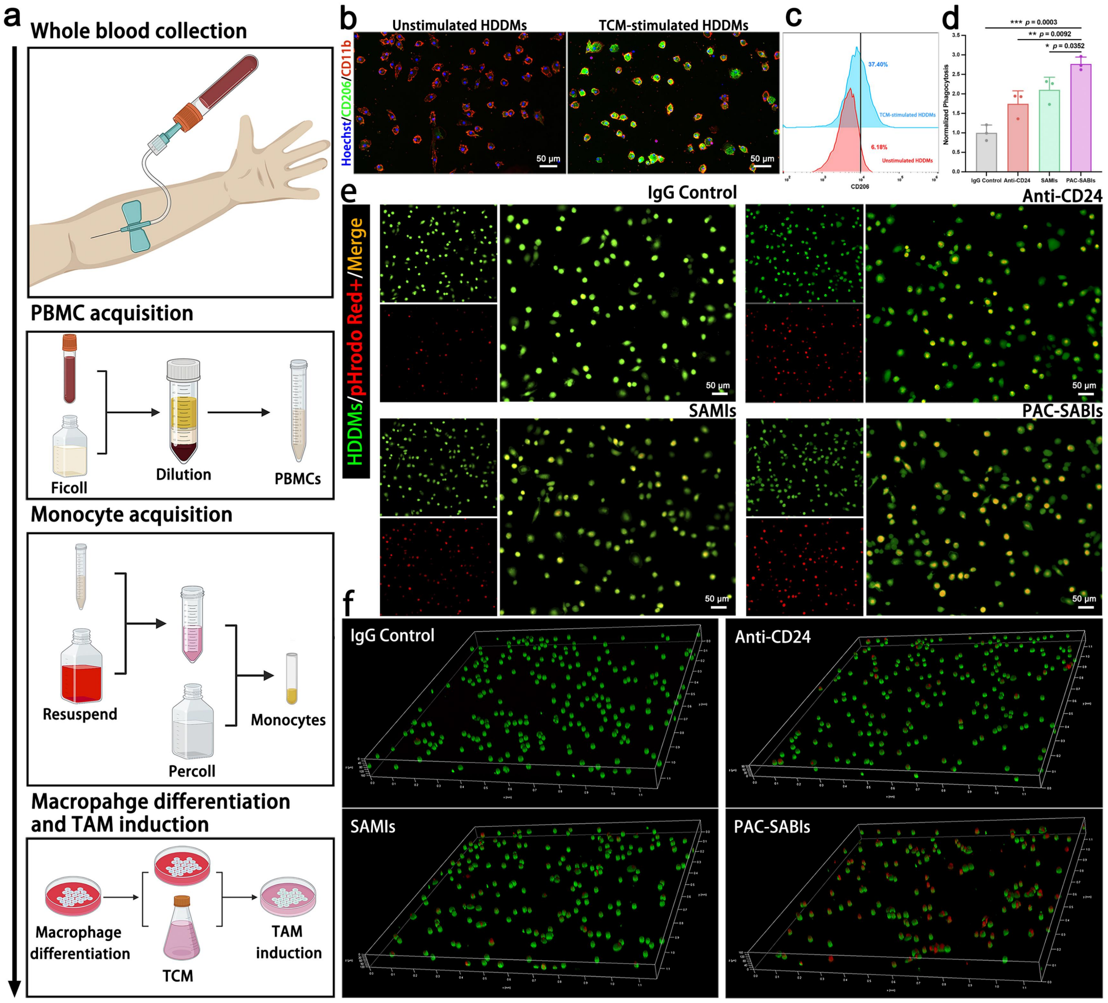

Supplementary Fig. 28 PAC-SABIs promote phagocytic clearance of cancer cells by HDDMs. **a** Schematic illustration of HDDMs generation and stimulation. Figure was created with BioRender.com and released under a Creative Commons Attribution-NonCommercial-NoDerivs 4.0 international license. **b** IF staining of CD11b and CD206 in HDDMs after stimulation with TCM for 48 h. Scale bar: 50 μm. Three independent experiments were performed. **c** Flow cytometry-based measurement of CD206 expression by HDDMs stimulated with TCM (blue) vs. cell medium control (red). **d** Normalized phagocytosis of MDA-MB-231 cells, in the presence of IgG control, anti-CD24 mAb, SAMIs and PAC-SABIs. The error bars represent the mean ± SD (n = 3 donors; $^{*}p < 0.05$, $^{**}p < 0.01$, $^{***}p < 0.001$; the p value was analyzed by a two-tailed unpaired Student's t-test). **e** Representative CLSM images of in vitro phagocytosis of MDA-MB-231 cells (pHrodo-red^{+}, red) by HDDMs (Calcein-AM, green). Scale bar: 50 μm. Three independent experiments were performed. **f** Representative 3D CLSM image reconstruction of in vitro phagocytosis of MDA-MB-231 cells (pHrodo-red^{+}, red) by HDDMs (Calcein-AM; green). Three

independent experiments were performed. Source data are provided as a Source Data file.

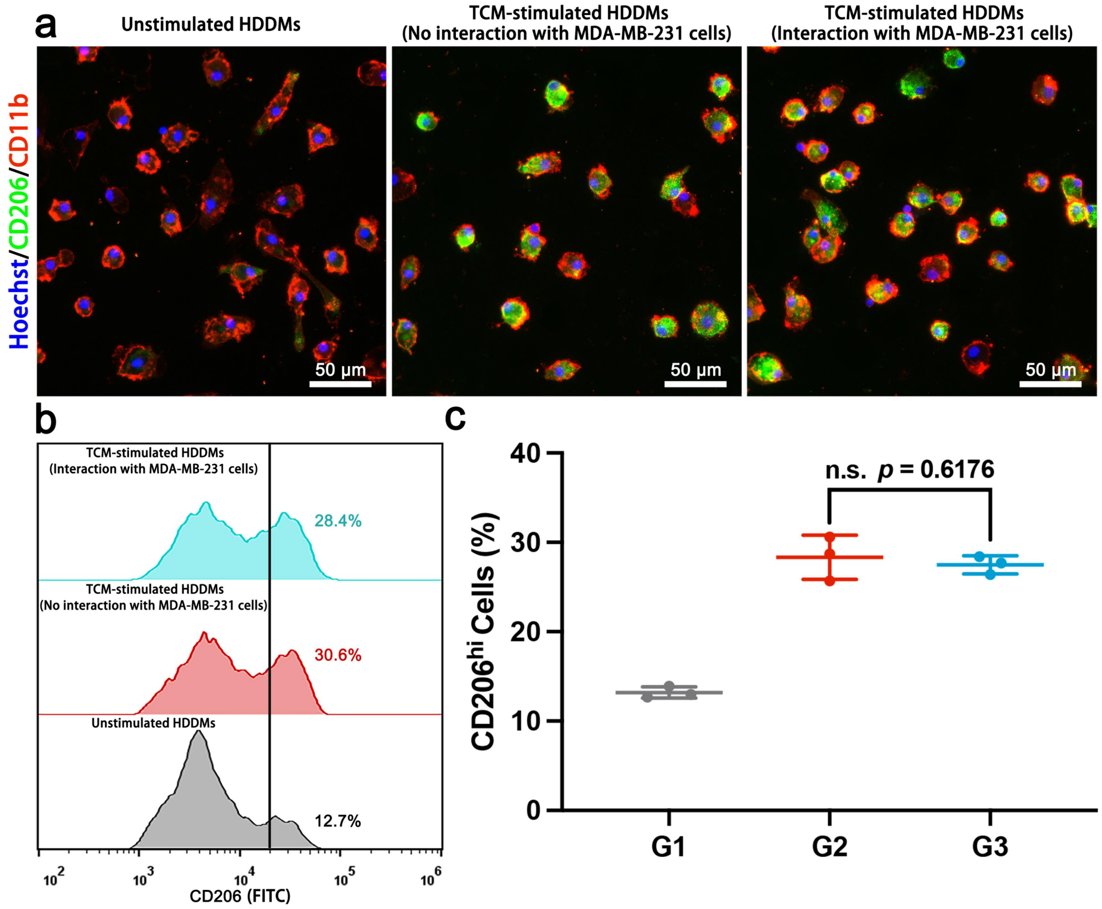

Supplementary Fig. 29 Transformation in TCM-stimulated HDDMs polarization. a IF staining of CD11b and CD206 in unstimulated HDDMs, TCM-stimulated HDDMs (No interaction with MDA-MB-231 cells), and TCM-stimulated HDDMs (Interaction with MDA-MB-231 cells). Scale bar: 50 μm. Three independent experiments were performed. **b** Flow cytometry-based measurement of CD206 expression by unstimulated HDDMs (grey), TCM-stimulated HDDMs (No interaction with MDA-MB-231 cells, red), and TCM-stimulated HDDMs (Interaction with MDA-MB-231 cells, blue). Three independent experiments were performed. **c** Quantification analysis of $CD206^{hi}$ macrophages after different treatments. G1 refers to unstimulated HDDMs; G2 refers to TCM-stimulated HDDMs (No interaction with MDA-MB-231 cells); G3 refers to TCM-stimulated HDDMs (Interaction with MDA-MB-231 cells). The error bars represent the mean ± SD (n = 3 donors; n.s. refers to no significance; the *p* value was analyzed by a two-tailed unpaired Student's t-test). Source data are provided as a Source Data file.

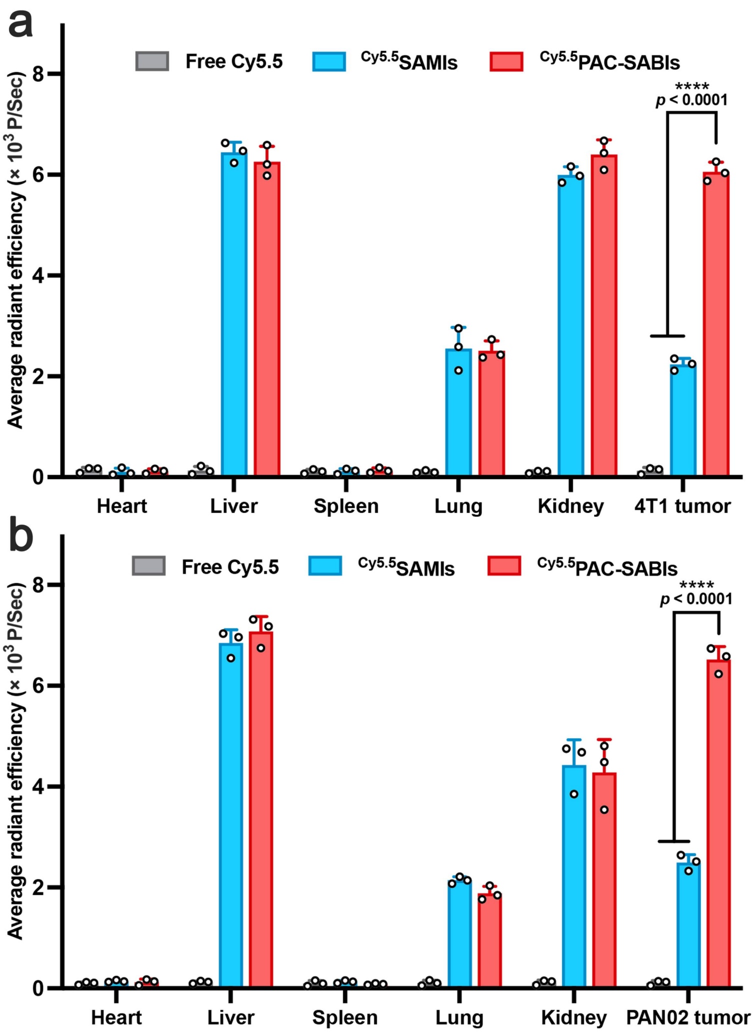

Supplementary Fig. 30 Quantification analysis of the ex vivo NIR fluorescence intensity in tumors and major organs. a Quantitative calculation of average fluorescence signal in 4T1 tumor and major organ area. The error bars represent the mean ± SD (n = 3 mice; $^{****}p < 0.0001$; the p value was analyzed by a two-tailed unpaired Student's t-test). **b** Quantitative calculation of average fluorescence signal in PAN02 tumor and major organ area. The error bars represent the mean ± SD (n = 3 mice; $^{****}p < 0.0001$; the p value was analyzed by a two-tailed unpaired Student's t-test). Source data are provided as a Source Data file.

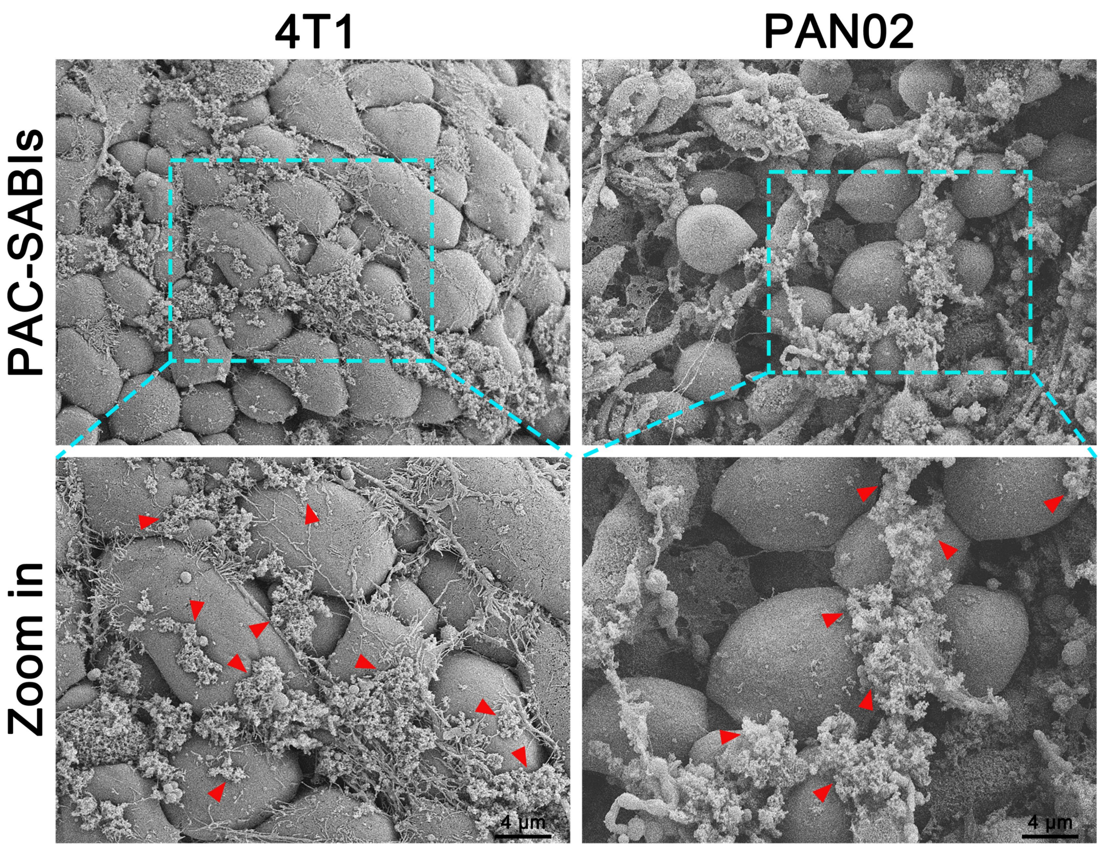

Supplementary Fig. 31 SEM images of PAC-SABIs accumulation within subcutaneous 4T1 and PAN02 xenograft. The red arrows point to the PAC-SABI nanofiber network scaffold. Scale bar: 4 μm. Three independent experiments were performed.

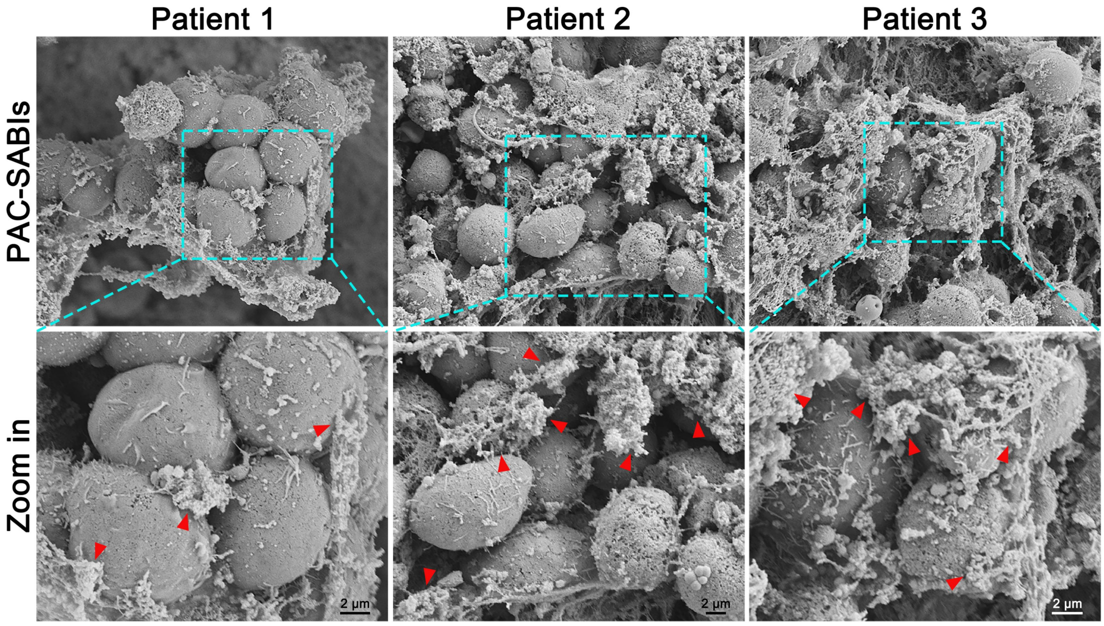

Supplementary Fig. 32 SEM images of PAC-SABIs accumulation within clinical BC samples.

The red arrows point to the PAC-SABI nanofiber network scaffold. Scale bar: 2 μm.

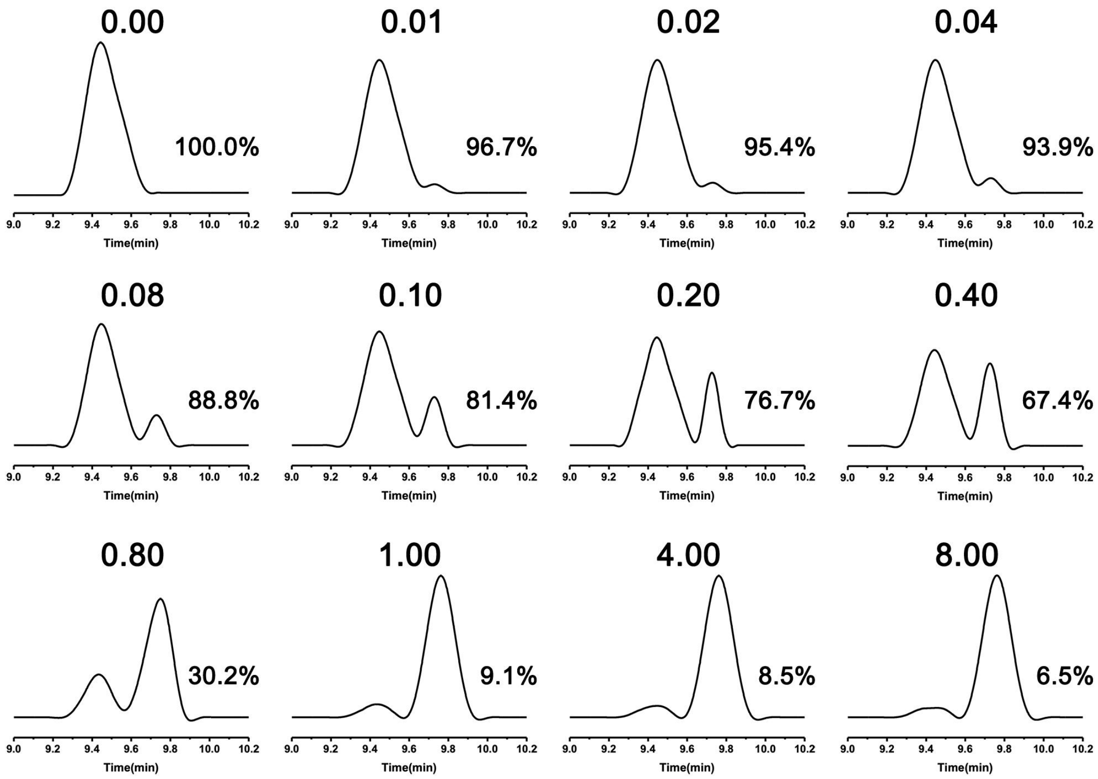

Supplementary Fig. 33 Stability of PAC-SABIs under different concentrations of ALP (U/mL) incubation conditions.

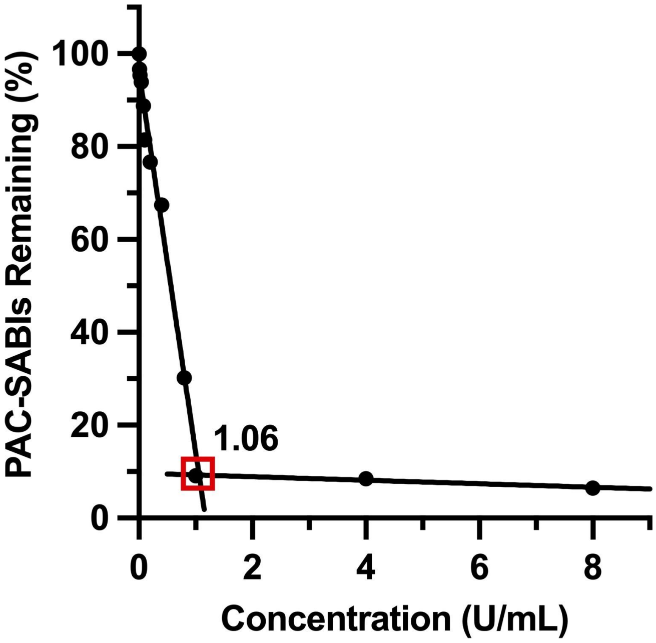

Supplementary Fig. 34 Two-piecewise linear regression model analysis for threshold value of ALP (U/mL). Source data are provided as a Source Data file.

Supplementary Table 2 Baseline clinical characteristics of the BC patients.

Characteristic	
Age, median (min, max), y	57 (31, 72)
Gender, n (%)	
Male	0 (0.0)
Female	22 (100.0)
Tumor type, n (%)	
Infiltrating ductal	18 (81.8)
Infiltrating lobular	3 (13.7)
Other	1 (4.5)
Tumor localization, n (%)	
Unilateral	21 (95.5)
Bilateral	1 (4.5)
ALP, median (min, max), U/L	64 (40, 146)

y, year; n, number; ALP, alkaline phosphatase; Normal range of ALP: 50-139 U/L.

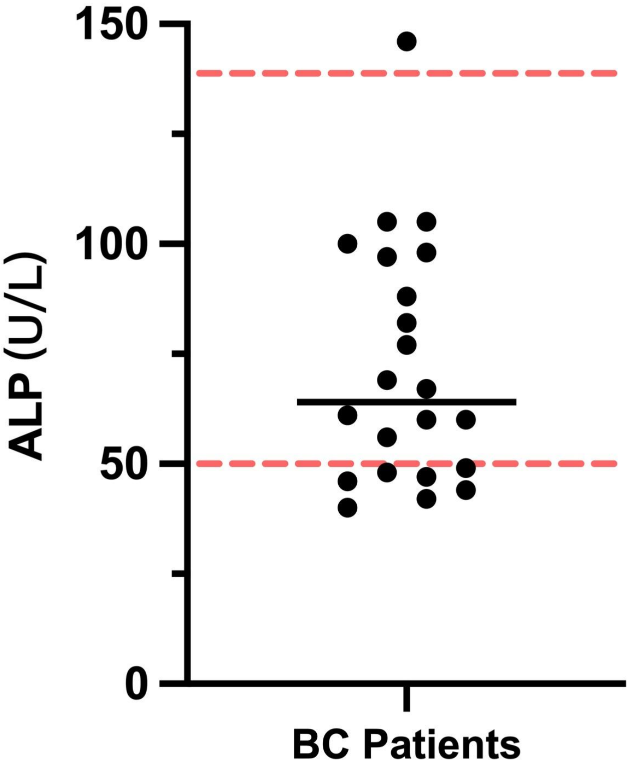

Supplementary Fig. 35 Serum ALP levels in clinical BC patients (U/L). The black line refers to the median (n = 22 patients). The upper and lower red dashed lines refer to the upper and lower limits of the normal ALP range (139 and 50 U/L, respectively). Source data are provided as a Source Data file.

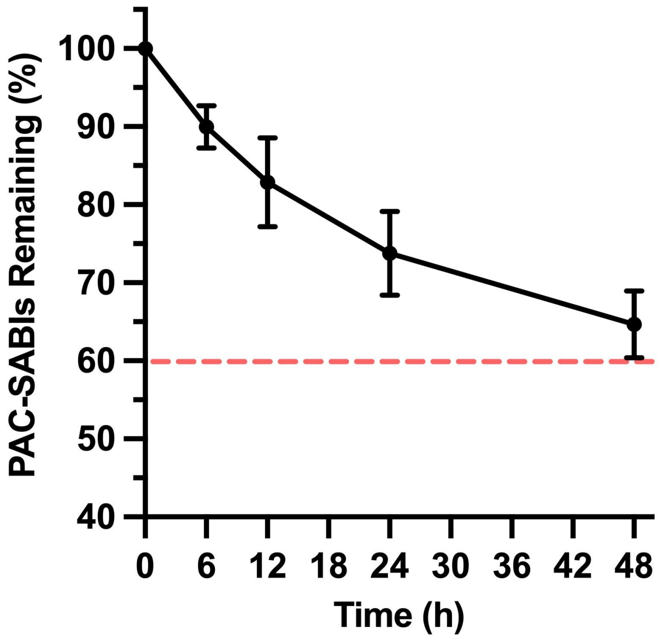

Supplementary Fig. 36 Stability of PAC-SABIs in serum of BC patients over time. The error bars represent the mean ± SD (n = 4 patients). The red dashed line refers to 60% residual PAC-SABIs. Source data are provided as a Source Data file.

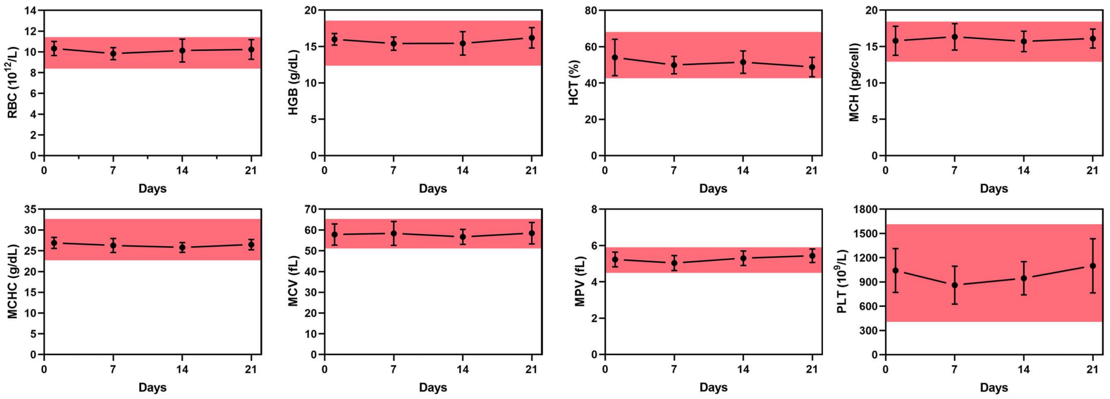

Supplementary Fig. 37 Complete blood count analyses. Complete blood count analyses including red blood cells (RBC), hemoglobin (HGB), hematocrit (HCT), mean corpuscular hemoglobin (MCH), mean corpuscular hemoglobin concentration (MCHC), mean corpuscular volume (MCV), mean platelet volume (MPV), platelet (PLT) after PAC-SABIs treatment. The error bars represent the mean ± SD (n = 3 mice). The red area refers to the normal range for each indicator. Source data are provided as a Source Data file.

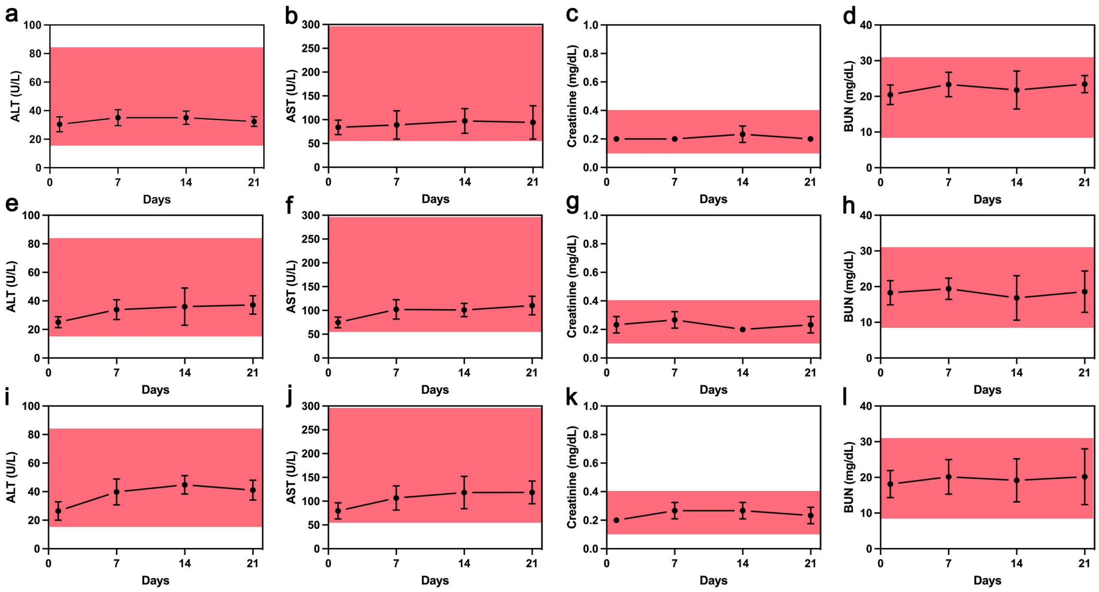

Supplementary Fig. 38 Hepatorenal toxicity analyses. a-d Blood biochemical analyses including alanine aminotransferase (ALT), aspartate aminotransferase (AST), creatinine, and blood urea nitrogen (BUN) after PAC-SABIs treatment (15 mg/kg). The error bars represent the mean ± SD (n = 3 mice). **e-h** Blood biochemical analyses including ALT, AST, Creatinine, and BUN after PAC-SABIs treatment (30 mg/kg). The error bars represent the mean ± SD (n = 3 mice). The red area refers to the normal range for each indicator. **i-l** Blood biochemical analyses including ALT, AST, Creatinine, and BUN after PAC-SABIs treatment (45 mg/kg). The error bars represent the mean ± SD (n = 3 mice). The red area refers to the normal range for each indicator. Source data are provided as a Source Data file.

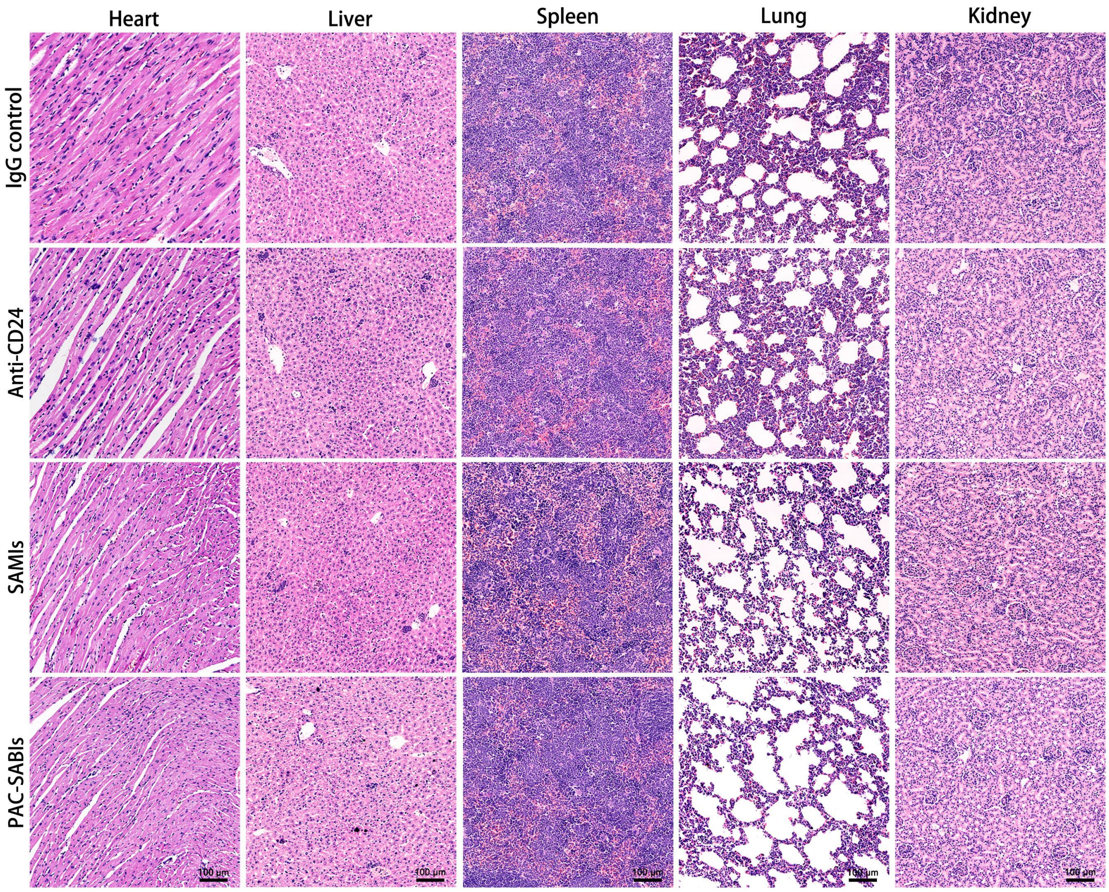

Supplementary Fig. 39 H&E staining. H&E staining of major organs (heart, liver, spleen, lungs, and kidneys) from each treatment group. Scale bar: 100 μm. Three independent experiments were performed.

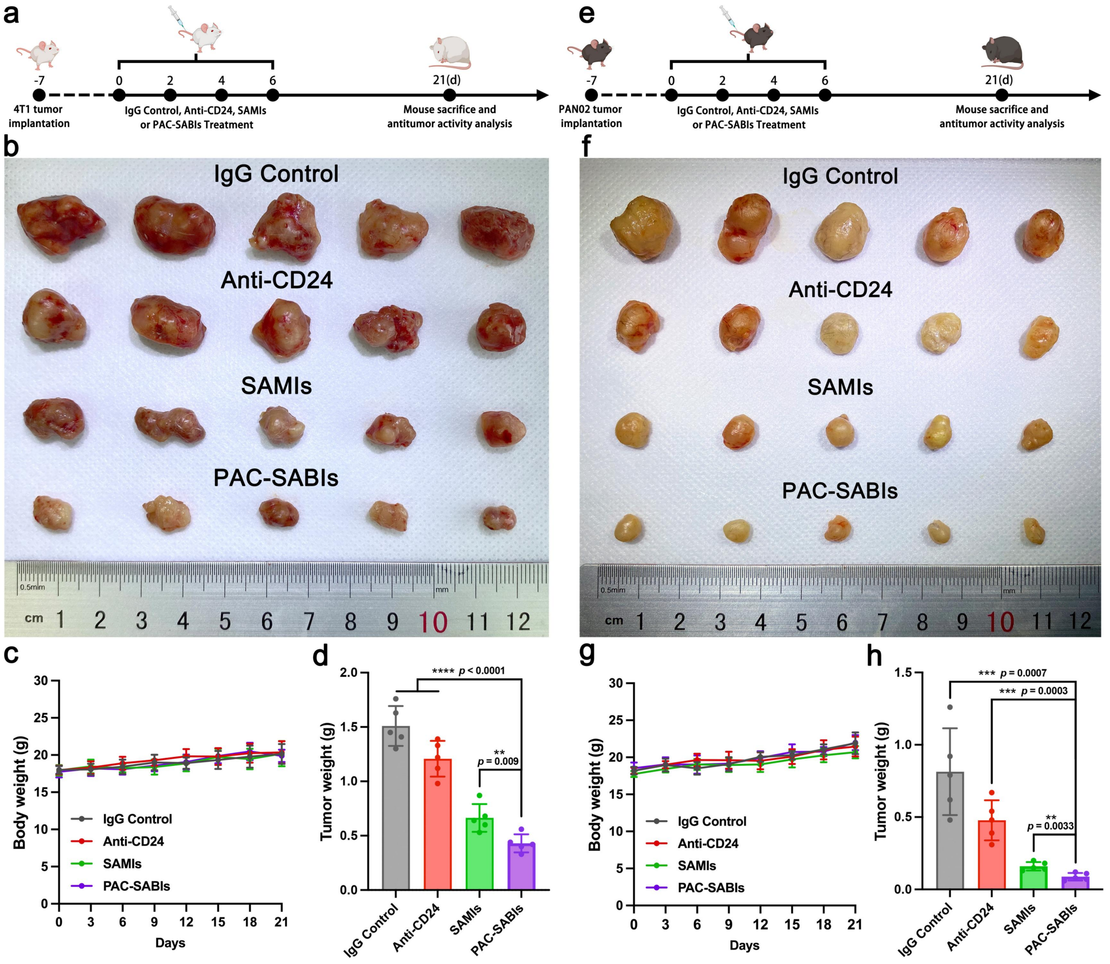

Supplementary Fig. 40 Antitumor efficacy of PAC-SABIs in vivo. **a** Scheme of the PAC-SABIs therapeutic strategy for 4T1 subcutaneous tumor. Figure was created with BioRender.com and released under a Creative Commons Attribution-NonCommercial-NoDerivs 4.0 international license. **b** Bright field images of excised 4T1 tumors. **c** Changes in 4T1 tumor-bearing mouse body weight during treatment. The error bars represent the mean ± SD (n = 5 mice). **d** 4T1 tumor weights of each group after treatment. The error bars represent the mean ± SD (n = 5 mice; $^{**}p < 0.01$, $^{****}p < 0.0001$; the p value was analyzed by a two-tailed unpaired Student's t-test). **e** Scheme of the PAC-SABIs therapeutic strategy for PAN02 subcutaneous tumor. Figure was created with BioRender.com and released under a Creative Commons Attribution-NonCommercial-NoDerivs 4.0 international license. **f** Bright field images of excised PAN02 tumors. **g** Changes in PAN02 tumor-bearing mouse body weight during treatment. The error bars represent the mean ± SD (n = 5 mice). **h** PAN02 tumor weights of each group after treatment. The error bars represent the mean ± SD (n = 5 mice; $^{**}p < 0.01$, $^{***}p < 0.001$; the p value

was analyzed by a two-tailed unpaired Student's t-test). Source data are provided as a Source Data file.

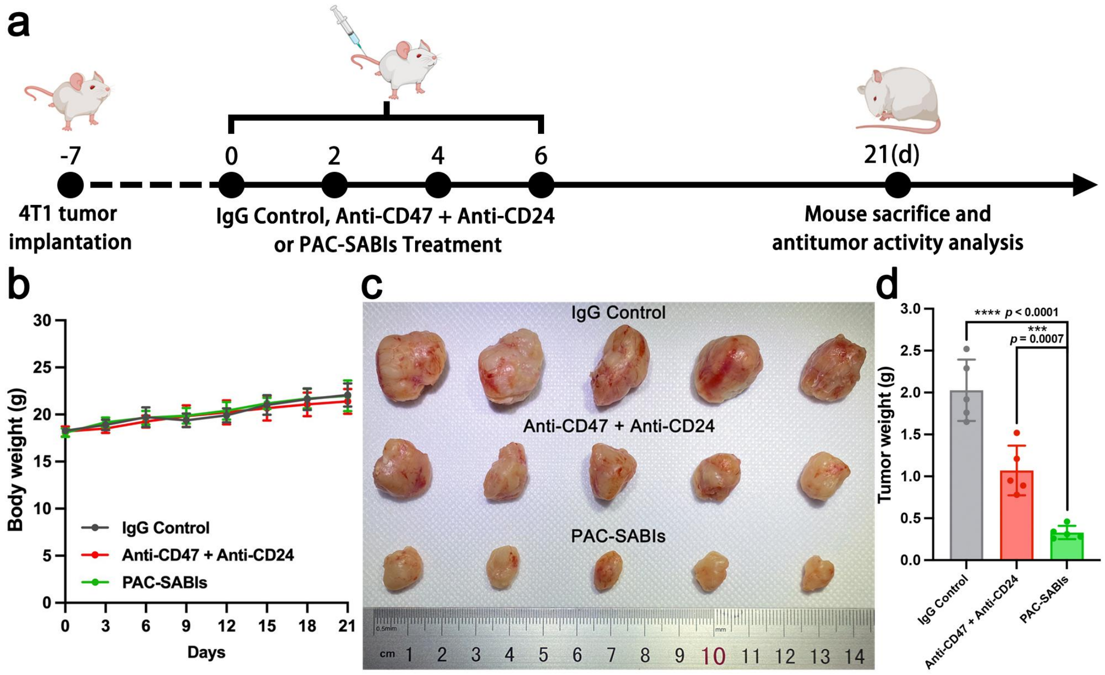

Supplementary Fig. 41 Comparison of in vivo antitumor effects of PAC-SABIs and dual antibody combination therapy. a Scheme of the therapeutic strategy for 4T1 subcutaneous tumor. Figure was created with BioRender.com and released under a Creative Commons Attribution-NonCommercial-NoDerivs 4.0 international license. **b** Changes in 4T1 tumor-bearing mouse body weight during treatment. The error bars represent the mean ± SD (n = 5 mice). **c** Bright field images of excised 4T1 tumors. **d** 4T1 tumor weights of each group after treatment. The error bars represent the mean ± SD (n = 5 mice; $^{***}p < 0.001$, $^{****}p < 0.0001$; the p value was analyzed by a two-tailed unpaired Student's t-test). Source data are provided as a Source Data file.

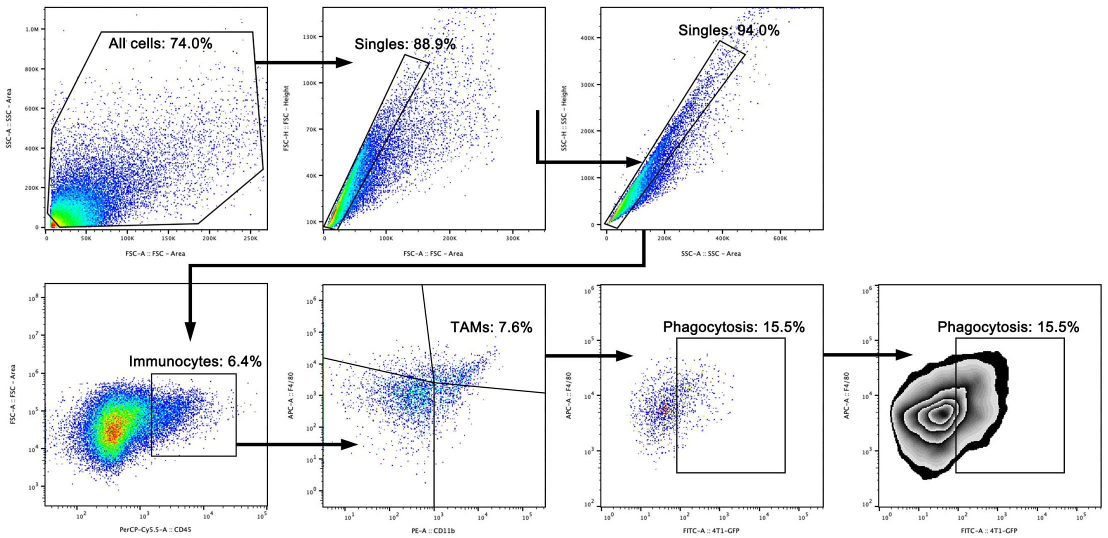

Supplementary Fig. 42 Gating strategy used for the evaluation of in vivo macrophage phagocytosis.

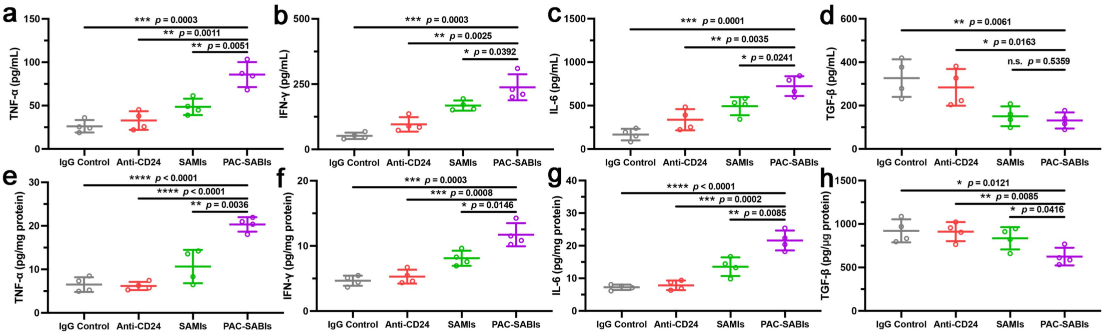

Supplementary Fig. 43 Cytokine levels in the plasma and 4T1 tumor tissue of mice. a-d Cytokine levels (TNF-α, IFN-γ, IL-6, and TGF-β) in the plasma of mice after different treatments determined using ELISA. The error bars represent the mean ± SD (n = 4 independent experiments; $^{*}p < 0.05$, $^{**}p < 0.01$, $^{***}p < 0.001$, n.s. refers to no significance; the *p* value was analyzed by a two-tailed unpaired Student's t-test). **e-h** Cytokine levels (TNF-α, IFN-γ, IL-6, and TGF-β) in the 4T1 tumor tissues after different treatments determined using ELISA. The error bars represent the mean ± SD (n = 4 independent experiments; $^{*}p < 0.05$, $^{**}p < 0.01$, $^{***}p < 0.001$, $^{****}p < 0.0001$; the *p* value was analyzed by a two-tailed unpaired Student's t-test). Source data are provided as a Source Data file.

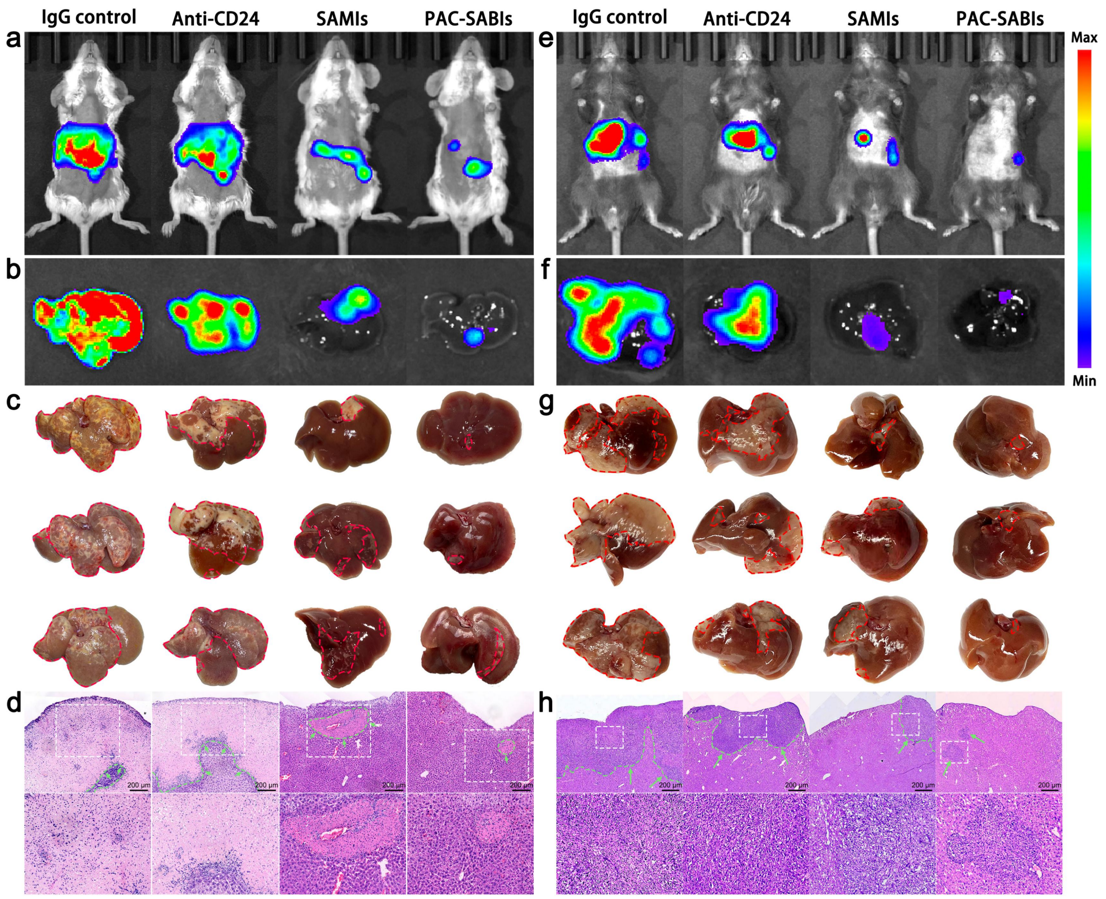

Supplementary Fig. 44 Inhibition on BC and PC liver metastasis in vivo. a Representative in vivo BLIs of mice bearing 4T1 liver metastasis receiving different treatments. **b** Representative ex vivo BLIs of isolated livers in each treatment group. **c** Bright field images of the 4T1 liver metastasis in each treatment group. **d** Representative H&E staining of the 4T1 liver metastasis in each treatment group. The green areas and arrows point to the liver metastases. Scale bar: 200 μm. Three independent experiments were performed. **e** Representative in vivo BLIs of mice bearing PAN02 liver metastasis receiving different treatments. **f** Representative ex vivo BLIs of isolated livers in each treatment group. **g** Bright field images of the PAN02 liver metastasis in each treatment group. **h** Representative H&E staining of the PAN02 liver metastasis in each treatment group. The green areas and arrows point to the liver metastases. Scale bar: 200 μm. Three independent experiments were performed.

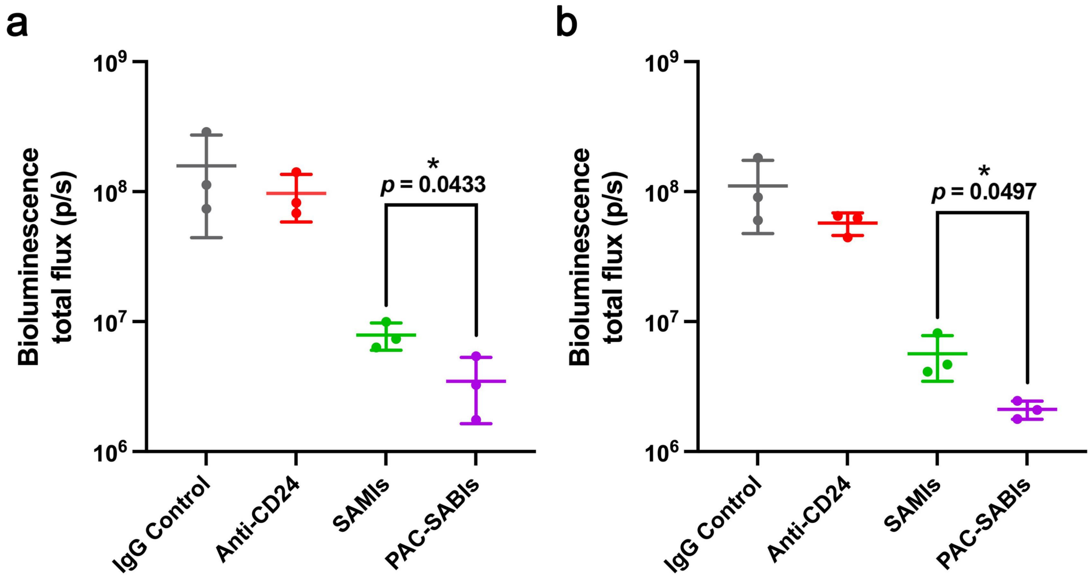

Supplementary Fig. 45 Quantification analysis of the in vivo BLI signals. a Quantification analysis of the BLI signals in BC liver metastasis after different treatments. The error bars represent the mean ± SD (n = 3 mice; $^*p < 0.05$; the p value was analyzed by a two-tailed unpaired Student's t-test). **b** Quantification analysis of the BLI signals in PC liver metastasis after different treatments. The error bars represent the mean ± SD (n = 3 mice; $^*p < 0.05$; the p value was analyzed by a two-tailed unpaired Student's t-test). Source data are provided as a Source Data file.

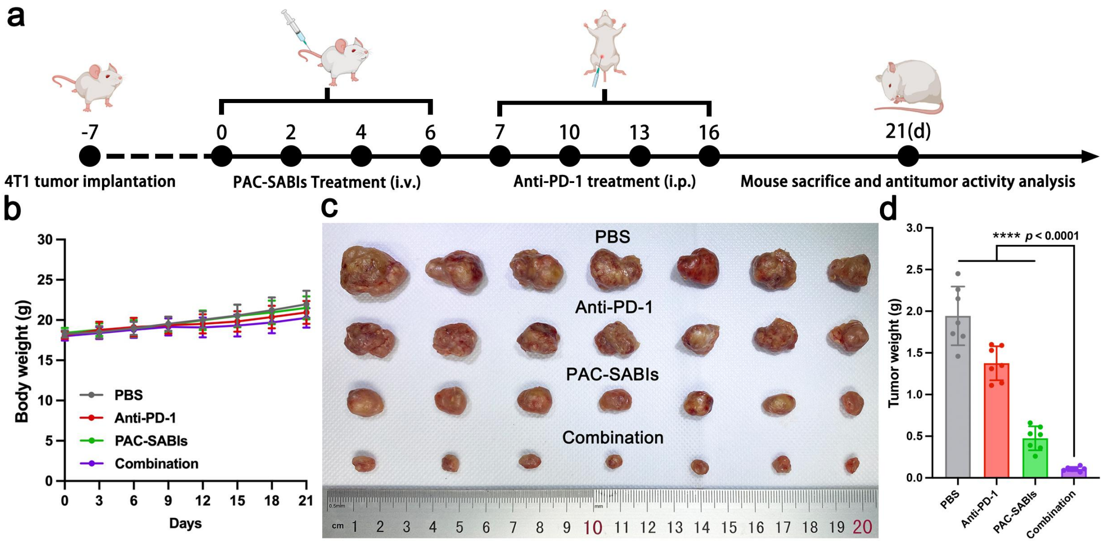

Supplementary Fig. 46 Enhanced anti-PD-1 therapy after phagocytosis modulation. a Scheme of the combination therapeutic strategy. Figure was created with BioRender.com and released under a Creative Commons Attribution-NonCommercial-NoDerivs 4.0 international license. **b** Changes in 4T1 tumor-bearing mouse body weight during treatment. The error bars represent the mean ± SD (n = 7 mice). **c** Bright field images of excised 4T1 tumors. **d** 4T1 tumor weights of each group after treatment. The error bars represent the mean ± SD (n = 7 mice; $^{****}p < 0.0001$; the p value was analyzed by a two-tailed unpaired Student's t-test). Source data are provided as a Source Data file.

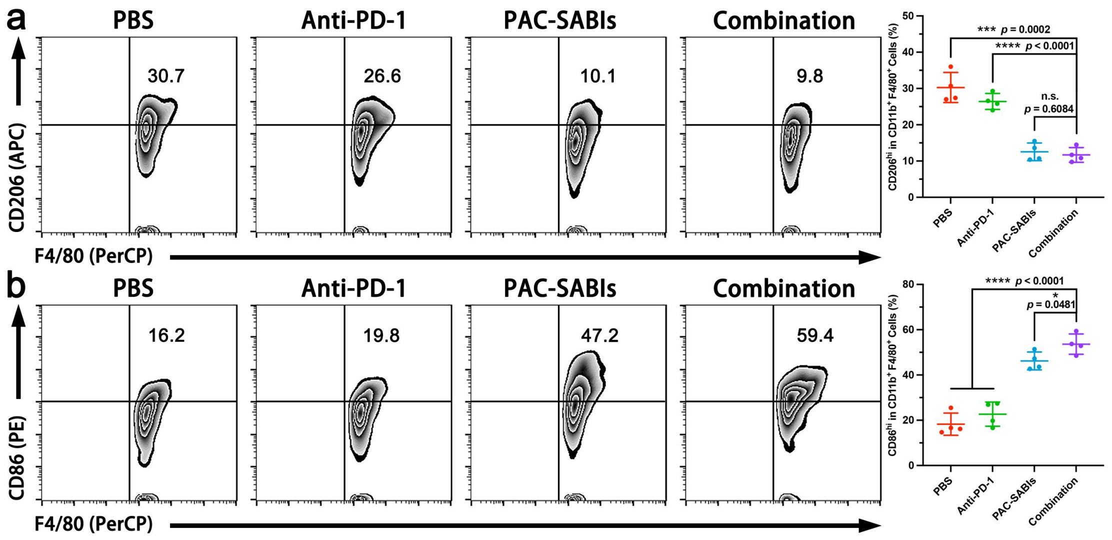

Supplementary Fig. 47 Macrophage polarization in vivo. a Representative flow cytometry plots and quantification analysis of $CD11b^{+}F4/80^{+}CD206^{hi}$ macrophages in 4T1 tumors after different treatments. The error bars represent the mean ± SD (n = 4 independent experiments; n.s. refers to no significance, $^{***}p < 0.001$, $^{****}p < 0.0001$; the p value was analyzed by a two-tailed unpaired Student's t-test). **b** Representative flow cytometry plots and quantification analysis of $CD11b^{+}F4/80^{+}CD86^{hi}$ macrophages in 4T1 tumors after different treatments. The error bars represent the mean ± SD (n = 4 independent experiments; $^{*}p < 0.05$, $^{****}p < 0.0001$; the p value was analyzed by a two-tailed unpaired Student's t-test). Source data are provided as a Source Data file.

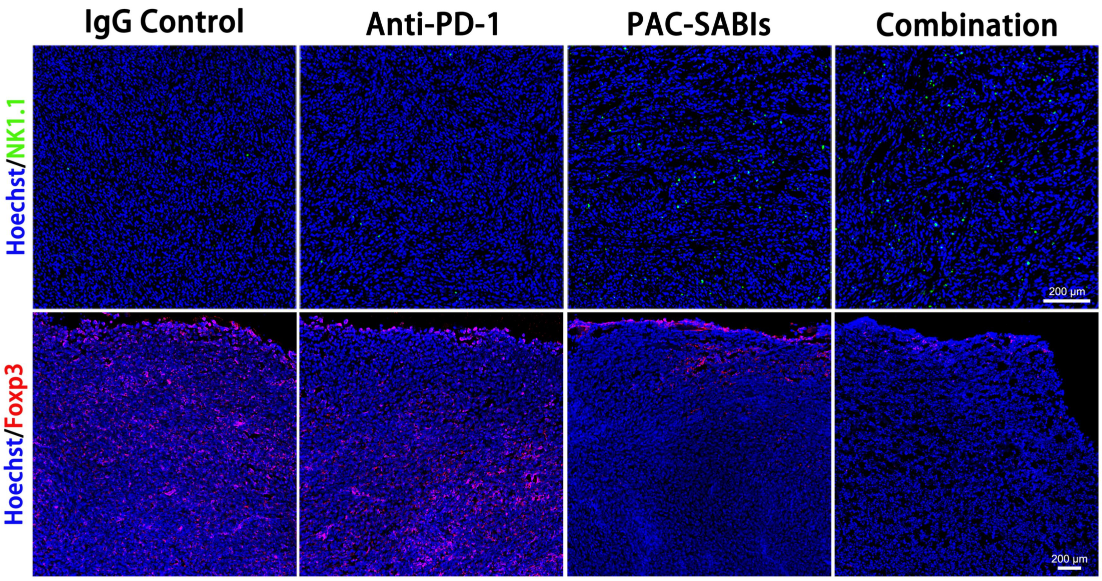

Supplementary Fig. 48 Representative IF staining of NK1.1$^+$ cells and Foxp3$^+$ T cells for the corresponding 4T1 tumor tissues after different treatments. Scale bar: 200 μm. Three independent experiments were performed.

Supplementary Table 3 MALDI-TOF mass spectrometry acquisition parameters.

Item	Parameter
Spectrum Size (mm)	0.4005
Motion Scanning Speed (mm/sec)	0.50
Averaged shots per spectrum	800
PulseFrequency (Hz)	1000.00
Detector Voltage (kV)	-0.5
Extraction Voltage (kV)	-3.2
Source Voltage (kV)	-20
Absolute Input Scale (V)	1.000
Sample Rate (ps)	800.00
Laser Pulse Current (Amps)	2.4
Laser Pulse Energy (μJ)	5
Warmup Delay (hh:mm:ss)	00:00:30
Pressure Target (Torr)	9E-07

Supplementary References

1. Tang, Z., Kang, B., Li, C., Chen, T. & Zhang, Z. GEPIA2: an enhanced web server for large-scale expression profiling and interactive analysis. *Nucleic Acids Res.* **47**, W556–W560 (2019).
