# Supplementary material for: An in-situ peptide-antibody self-assembly to block CD47 and CD24 signaling enhances macrophage-mediated phagocytosis and anti-tumor immune responses
Source: Nat Commun. 2024 Jul 6;15:5670. doi: 10.1038/s41467-024-49825-6 (PMC11227529; doi:10.1038/s41467-024-49825-6)
Supplement: Supplementary file 3 — Reporting Summary [file 41467_2024_49825_MOESM3_ESM.pdf]

nature portfolio

Corresponding author(s): Yanbin Cai

Last updated by author(s): Jun 18, 2024

Reporting Summary

Nature Portfolio wishes to improve the reproducibility of the work that we publish. This form provides structure for consistency and transparency in reporting. For further information on Nature Portfolio policies, see our Editorial Policies and the Editorial Policy Checklist.

Statistics

For all statistical analyses, confirm that the following items are present in the figure legend, table legend, main text, or Methods section.

n/a	Confirmed	
☐	☒	The exact sample size (*n*) for each experimental group/condition, given as a discrete number and unit of measurement
☐	☒	A statement on whether measurements were taken from distinct samples or whether the same sample was measured repeatedly
☐	☒	The statistical test(s) used AND whether they are one- or two-sided<br>*Only common tests should be described solely by name; describe more complex techniques in the Methods section.*
☐	☒	A description of all covariates tested
☐	☒	A description of any assumptions or corrections, such as tests of normality and adjustment for multiple comparisons
☐	☒	A full description of the statistical parameters including central tendency (e.g. means) or other basic estimates (e.g. regression coefficient) AND variation (e.g. standard deviation) or associated estimates of uncertainty (e.g. confidence intervals)
☐	☒	For null hypothesis testing, the test statistic (e.g. *F*, *t*, *r*) with confidence intervals, effect sizes, degrees of freedom and *P* value noted<br>*Give P values as exact values whenever suitable.*
☒	☐	For Bayesian analysis, information on the choice of priors and Markov chain Monte Carlo settings
☒	☐	For hierarchical and complex designs, identification of the appropriate level for tests and full reporting of outcomes
☒	☐	Estimates of effect sizes (e.g. Cohen's *d*, Pearson's *r*), indicating how they were calculated

Our web collection on statistics for biologists contains articles on many of the points above.

Software and code

Policy information about availability of computer code

Data collection	Elite P3500 Semi-preparative Integrated Liquid Chromatography System (Elite Analytical Instruments, China) for HPLC purification, QuanTOF I system (Intelligene Biosystems, China) for mass spectrometry analysis, Perkin Elmer LS-55 fluorescence spectrophotometer (Perkin Elmer, USA) for fluorescence assays, CD spectropolarimeter (Applied Photophysics Ltd, UK) for CD spectroscopy, Nicolet 6700 FTIR spectrometer (Thermo Fisher Scientific, USA) for FTIR spectroscopy, SYNERGY H1 multifunctional microplate reader (BioTek, USA) for absorbance value measurement, JEOL JEM-2100F field emission transmission electron microscope for TEM characterization, Zeiss Ultra 55 SEM (Carl Zeiss, Germany) for SEM characterization, Pannoramic MIDI digital slide scanner (3DHISTECH, Hungary) for section scanning, Nikon AX confocal laser microscope (Nikon, Japan) for confocal imaging, Sony SA3800 Flow Cytometer (Sony Biotechnology, Japan) or CytoFLEX (Beckman, USA) for flow cytometry, IVIS system (Perkin Elmer, USA) for in vivo fluorescence imaging.
Data analysis	Microsoft office 2021, GraphPad Prism version 10.2.1, SPSS version 22.0, FIJI (FIJI is just imageJ) version 2.1.0, Chromsoft Workstation, QuanTOF version 1.0.59375, Gen5 version 3.08, CaseViewer version 2.4.0.119028, NIS-Elements version 5.4, Flowjo version 10.4, RStudio version 2022.02, Chemdraw version 16.0.1.4, Livingimage version 4.3.1, DichroWeb server

For manuscripts utilizing custom algorithms or software that are central to the research but not yet described in published literature, software must be made available to editors and reviewers. We strongly encourage code deposition in a community repository (e.g. GitHub). See the Nature Portfolio guidelines for submitting code & software for further information.

Data

Policy information about availability of data

All manuscripts must include a data availability statement. This statement should provide the following information, where applicable:

- Accession codes, unique identifiers, or web links for publicly available datasets
- A description of any restrictions on data availability
- For clinical datasets or third party data, please ensure that the statement adheres to our policy

RNA-seq data that support the findings of this study have been deposited in the NCBI Sequence Read Archive (SRA, http://www.ncbi.nlm.nih.gov/sra) under the BioProject ID PRJNA1124390. RNA-seq data based on the TCGA and Genotype-Tissue Expression (GTEx) projects, obtained from the Gene Expression Profiling Interactive Analysis 2 (GEPIA2, http://gepia2.cancer-pku.cn) database, were used to investigate the expression of CD47 and CD24 in tumor and paired normal tissue, and to conduct survival analysis. The remaining data generated in this study are available within the article and its Supplementary Information files. Data generated in this study are provided in the Source Data file. Source data are provided with this paper.

Research involving human participants, their data, or biological material

Policy information about studies with human participants or human data. See also policy information about sex, gender (identity/presentation), and sexual orientation and race, ethnicity and racism.

Reporting on sex and gender	Sex was not a defining factor in participant selection for this study. The breast cancer patients in our study were female by nature of the disease. The healthy volunteers and pancreatic cancer patients in our study were male, not due to any sex bias but as a result of random selection.
Reporting on race, ethnicity, or other socially relevant groupings	All participants included in this research were Chinese, with the ethnicity being the Asian subgroup.
Population characteristics	No direct human research participants were involved in this study. Human peripheral blood, breast cancer and pancreatic cancer specimens were obtained.
Recruitment	We obtained 3 whole blood samples from 3 healthy volunteers (aged 26, 27, and 30 years old respectively), 4 whole blood samples from 4 breast cancer patients (aged 48, 54, 57, and 64 years old respectively), biopsies from 2 different breast cancer patients (2 biopsies each for patient 1 and 2, aged 42 and 45 years old respectively), radical mastectomies with tumor-only samples form 4 breast cancer patients (aged 45, 48, 55,and 60 years old respectively), 1 pancreaticoduodenectomy with tumor-only sample from 1 pancreatic cancer patient (aged 58 years old). All breast cancer and pancreatic cancer patients had lesions that were visible on preoperative ultrasound or MRI and were later confirmed pathologically to be cancer.
Ethics oversight	Approvals were obtained from the institutional review board of Nanfang Hospital and Guangdong Provincial People's Hospital of Southern Medical University (NFEC-2023-309, KY2023-179-01, and KY2023-1143-02). Human peripheral blood, BC and PC specimens were sourced, and the appropriate informed consent was obtained for all sample donors.

Note that full information on the approval of the study protocol must also be provided in the manuscript.

Field-specific reporting

Please select the one below that is the best fit for your research. If you are not sure, read the appropriate sections before making your selection.

☒ Life sciences ☐ Behavioural & social sciences ☐ Ecological, evolutionary & environmental sciences

For a reference copy of the document with all sections, see nature.com/documents/nr-reporting-summary-flat.pdf

Life sciences study design

All studies must disclose on these points even when the disclosure is negative.

Sample size	Sample sizes were not statistically pre-determined but were based on the general requirements of assays. We followed the sample size selections according to the related research and experimental assays that were cited in the main text. For in vitro experiments such as flow cytometry, at least three samples were used per group for minimal statistics requirements. For in vivo studies, the sample size was determined to be sufficient to obtain the statistical difference between groups.
Data exclusions	No data were excluded from the analyses.
Replication	At least three independent experiments were performed. The biological replicates were n=3, 5 or 7, respectively. All attempts at replication were successful.
Randomization	All samples were allocated to groups randomly.
Blinding	Investigators were not blinded as they designed the experiments and also performed the investigations. Therefore, the investigators

maintained awareness of the experimental conditions throughout the data acquisition and analysis process. However, the investigators were unbiased in both outcome assessment and conclusion drawing. Critical experiments were analyzed by at least two independent investigators for unbiased conclusion.

Reporting for specific materials, systems and methods

We require information from authors about some types of materials, experimental systems and methods used in many studies. Here, indicate whether each material, system or method listed is relevant to your study. If you are not sure if a list item applies to your research, read the appropriate section before selecting a response.

Materials & experimental systems

n/a	Involved in the study
	☒ Antibodies
	☒ Eukaryotic cell lines
☒	☐ Palaeontology and archaeology
	☒ Animals and other organisms
☒	☐ Clinical data
☒	☐ Dual use research of concern
☒	☐ Plants

Methods

n/a	Involved in the study
☒	☐ ChIP-seq
	☒ Flow cytometry
☒	☐ MRI-based neuroimaging

Antibodies

Antibodies used

The following antibodies have been applied in this study:
anti-F4/80 antibody (CI:A3-1, Abcam, ab6640, 10 μg/ml for IF)
anti-CK19 antibody (EP1580Y, Abcam, ab52625, 2 μg/ml for IF)
anti-CD8α antibody (208, Invitrogen, MA5-29682, 1:100 for IF)
goat anti-rat IgG H&L (Abcam, ab150167, 1:1000 for IF)
goat anti-rabbit IgG H&L (Abcam, ab150077, 1:1000 for IF)
anti-CD24 antibody (EPR26528-17, Abcam, ab290730, 1:50 for IF)
anti-mouse CD24 antibody (M1/69, BioXcell, BE0360)
anti-human CD24 antibody (SN3, Novus Biologics, NB100-64861)
anti-CD47 antibody (EPR24922-21, Abcam, ab300124, 1:400 for IF)
anti-mouse CD47 antibody (MIAP301, BioXcell, BE0270)
anti-mouse PD-1 (29F.1A12™, BioXcell, BE0273)
anti-CSF1R antibody (AFS98, BioXcell, BE0213)
anti-NKR-P1C antibody (EPR22990-31, Abcam, ab289542, 1:50 for IF)
anti-Foxp3 antibody (Affinity Biosciences, AF6544, 1:100 for IF)
anti-mouse CD45 antibody (30-F11, Elabscience, E-AB-F1136J, 1:200 for Flow Cyt)
anti-mouse CD11b antibody (M1/70, Biolegend, 101209, 1:200 for Flow Cyt)
anti-mouse F4/80 antibody (BM8, Biolegend, 123116, 1:200 for Flow Cyt)
anti-mouse CD206 antibody (C068C2, Biolegend, 141703, 1:200 for Flow Cyt)
anti-mouse CD86 antibody (A17199A, Biolegend, 159219, 1:200 for Flow Cyt)
anti-mouse CD3 antibody (17A2, BDbioscience, 555274, 1:200 for Flow Cyt)
anti-mouse CD4 antibody (RM4-5, BDbioscience, 553051, 1:200 for Flow Cyt)
anti-mouse CD8 antibody (53-6.7, BDbioscience, 553033, 1:200 for Flow Cyt)
anti-mouse Foxp3 antibody (R16-715, BDbioscience, 563101, 1:200 for Flow Cyt)
anti-human IgG Fc antibody (M1310G05, Biolegend, 410711, 1:200 for Flow Cyt)
anti-mouse IgG1 Fc antibody (MOPC-21 Biolegend, 400121, 1:200 for Flow Cyt)

Validation

All antibodies are commercially available and have been validated by the manufacturers:

anti-F4/80 antibody (CI:A3-1, Abcam, ab6640, https://www.abcam.com/products/primary-antibodies/f480-antibody-cia3-1-macrophage-marker-ab6640.html)

anti-CK19 antibody (EP1580Y, Abcam, ab52625, https://www.abcam.com/products/primary-antibodies/cytokeratin-19-antibody-ep1580y-cytoskeleton-marker-ab52625.html)

anti-CD8α antibody (208, Invitrogen, MA5-29682, https://www.thermofisher.com/antibody/product/CD8-alpha-Antibody-clone-208-Recombinant-Monoclonal/MA5-29682)

anti-CD24 antibody (EPR26528-17, Abcam, ab290730, https://www.abcam.com/products/primary-antibodies/cd24-antibody-epr26528-17-ab290730.html)

anti-mouse CD24 antibody (M1/69, BioXcell, BE0360, https://bioxcell.com/invivomab-anti-mouse-cd24-be0360)

anti-human CD24 antibody (SN3, Novus Biologics, NB100-64861, https://www.novusbio.com/products/cd24-antibody-sn3_nb100-64861)

anti-CD47 antibody (EPR24922-21, Abcam, ab300124, https://www.abcam.com/products/primary-antibodies/cd47-antibody-epr24922-21-ab300124.html)

anti-mouse CD47 antibody (MIAP301, BioXcell, BE0270, https://bioxcell.com/invivomab-anti-mouse-cd47-iap-be0270)

anti-mouse PD-1 (29F.1A12™, BioXcell, BE0273, https://bioxcell.com/invivomab-anti-mouse-pd-1-cd279-be0273)

anti-CSF1R antibody (AFS98, BioXcell, BE0213, https://bioxcell.com/invivomab-anti-mouse-csf1r-cd115-be0213)

anti-NKR-P1C antibody (EPR22990-31, Abcam, ab289542, https://www.abcam.com/products/primary-antibodies/nkr-p1c-antibody-epr22990-31-ab289542.html)

anti-Foxp3 antibody (Affinity Biosciences, AF6544, https://www.affbiotech.com/goods-17630-AF6544-Foxp3_Antibody.html)

anti-mouse CD45 antibody (30-F11, Elabscience, E-AB-F1136J, https://www.elabscience.com/p-percp_cyanine5.5_anti_mouse_cd45_antibody_30_f11_-151477.html)

anti-mouse CD11b antibody (M1/70, Biolegend, 101209, https://www.biolegend.com/nl-be/products/pe-cyanine5-anti-mouse-human-cd11b-antibody-350)

anti-mouse F4/80 antibody (BM8, Biolegend, 123116, https://www.biolegend.com/en-us/products/apc-anti-mouse-f4-80-antibody-4071)

anti-mouse CD206 antibody (C068C2, Biolegend, 141703, https://www.biolegend.com/en-us/products/fitc-anti-mouse-cd206-mmr-antibody-7318)

anti-mouse CD86 antibody (A17199A, Biolegend, 159219, https://www.biolegend.com/en-us/products/fitc-anti-mouse-cd86-antibody-24674)

anti-mouse CD3 antibody (17A2, BDbioscience, 555274, https://www.bdbiosciences.com/en-us/products/reagents/flow-cytometry-reagents/research-reagents/single-color-antibodies-ruo/fitc-rat-anti-mouse-cd3-molecular-complex.555274)

anti-mouse CD4 antibody (RM4-5, BDbioscience, 553051, https://www.bdbiosciences.com/en-us/products/reagents/flow-cytometry-reagents/research-reagents/single-color-antibodies-ruo/apc-rat-anti-mouse-cd4.553051)

anti-mouse CD8 antibody (53-6.7, BDbioscience, 553033, https://www.bdbiosciences.com/en-us/products/reagents/flow-cytometry-reagents/research-reagents/single-color-antibodies-ruo/pe-rat-anti-mouse-cd8a.553033)

anti-mouse Foxp3 antibody (R16-715, BDbioscience, 563101, https://www.bdbiosciences.com/en-us/products/reagents/flow-cytometry-reagents/research-reagents/single-color-antibodies-ruo/pe-rat-anti-mouse-foxp3.563101)

anti-human IgG Fc antibody (M1310G05, Biolegend, 410711, https://www.biolegend.com/en-us/products/apc-anti-human-igg-fc-11935)

anti-mouse IgG1 Fc antibody (MOPC-21 Biolegend, 400121, https://www.biolegend.com/en-us/products/apc-mouse-igg1-kappa-isotype-ctrl-fc-3034)

Eukaryotic cell lines

Policy information about cell lines and Sex and Gender in Research

Cell line source(s)	Murine breast cancer 4T1 cell line (CRL-2539), murine pancreatic cancer PAN02 cell line (CRL-2553), human breast cancer MDA-MB-231 cell line (CRM-HTB-26), murine macrophage cell line RAW264.7 (TIB-71), and human macrophage cell line THP-1 (TIB-202) were purchased from the American Type Culture Collection (ATCC). Primary human donor-derived macrophages were generated from venous blood of healthy volunteers (male). Bone marrow-derived macrophages were extracted from the femur and tibia of C57BL/6 mice (male).
Authentication	The cell lines were certified by the manufacturers (surface markers, morphology).
Mycoplasma contamination	All of the cell lines were tested negative for mycoplasma.
Commonly misidentified lines (See ICLAC register)	No commonly misidentified lines were used.

Animals and other research organisms

Policy information about studies involving animals; ARRIVE guidelines recommended for reporting animal research, and Sex and Gender in Research

Laboratory animals	C57BL/6 mice (6 weeks old) and BALB/c mice (6 weeks old) were purchased from Sijiajingda Biotechnology Co. The living environment of animals were maintained at a temperature of 22 °C and relative humidity range between 30-70 % with a 12 h light/dark cycle, with free access to standard food and water.
Wild animals	The study did not involve wide animals.
Reporting on sex	C57BL/6 mice (male), BALB/c mice (female). Although we have used single-sex animals in our research to establish the breast and pancreatic cancer models, we think that the research results are not exclusive to a particular sex, but are universally applicable.
Field-collected samples	The study did not involve samples collected from field.
Ethics oversight	Experiments were performed in agreement with the Animal Experimentation Ethics Committee of Southern Medical University (LAEC-2022-077 and LAEC-2024-015).

Note that full information on the approval of the study protocol must also be provided in the manuscript.

Plants

Seed stocks	The study did not involve plants.
Novel plant genotypes	The study did not involve plants.
Authentication	The study did not involve plants.

Flow Cytometry

Plots

Confirm that:

- [x] The axis labels state the marker and fluorochrome used (e.g. CD4-FITC).
- [x] The axis scales are clearly visible. Include numbers along axes only for bottom left plot of group (a 'group' is an analysis of identical markers).
- [x] All plots are contour plots with outliers or pseudocolor plots.
- [x] A numerical value for number of cells or percentage (with statistics) is provided.

Methodology

Sample preparation	The in vitro phagocytosis assays described in this study were performed by co-culture GFP+ 4T1 cells and macrophages at a ratio of 100,000 target cells to 50,000 macrophages for 120 min in a humidified, 5 % CO_2 incubator at 37 °C in ultra-low-attachment 96-well U-bottom plates (Corning, USA) in serum-free IMDM. 4T1 cells with endogenous fluorescence were harvested from plates using TrypLE Express (Life Technologies, Poland) and treated with PAC-SABIs for 120 min prior to co-culture. After co-culture, phagocytosis assays were stopped by placing plates on ice, centrifuged at 400 g for 5 min at 4 °C and stained with anti-CD11b to identify macrophages. Assays were analyzed by flow cytometry on a Sony SA3800 Flow Cytometer (Sony Biotechnology, Japan) or a CytoFLEX (Beckman, USA).<br>For the in vivo phagocytosis analysis, 4T1 tumors were cut into small pieces and homogenized in cold staining buffer to form single cell suspensions in the presence of digestive enzyme. Cells were stained with different fluorescence-labelled anti-CD45, anti-CD11b, and anti-F4/80 antibodies following the manufacturer's instructions. Assays were analyzed by flow cytometry on a Sony SA3800 Flow Cytometer (Sony Biotechnology, Japan) or a CytoFLEX (Beckman, USA).
Instrument	Sony SA3800 Flow Cytometer (Sony Biotechnology, Japan) or CytoFLEX (Beckman, USA)
Software	Flowjo 10.4
Cell population abundance	No cell sorting was performed.

Gating strategy	In vitro phagocytosis was measured as the number of CD11b+, GFP+ macrophages, quantified as a percentage of the total CD11b+ macrophages. In vivo phagocytosis was measured as the percentage of CD11b+, F4/80+ TAMs that were also GFP+.

☒ Tick this box to confirm that a figure exemplifying the gating strategy is provided in the Supplementary Information.
